# Nebulized Recombinant Tissue Plasminogen Activator (rt-PA) for Acute COVID-19-Induced Respiratory Failure: An Exploratory Proof-of-Concept Trial

**DOI:** 10.3390/jcm12185848

**Published:** 2023-09-08

**Authors:** Pratima Chowdary, Banwari Agarwal, Maria Rita Peralta, Sanjay Bhagani, Simon Lee, James Goldring, Marc Lipman, Emal Waqif, Mark Phillips, Helen Philippou, Jonathan H. Foley, Nicola J. Mutch, Robert A. S. Ariëns, Kathleen A. Stringer, Federico Ricciardi, Marie Watissée, Derralynn Hughes, Amit Nathwani, Anne Riddell, David Patch, Jim Buckley, Mark De Neef, Rahul Dimber, Cecilia Diaz-Garcia, Honey Patel, Aarti Nandani, Upuli Dissanayake, Nick Chadwick, Ahmed A. A. M. M. Alkhatip, Peter Watkinson, Eamon Raith, Suveer Singh, Tony Wolff, Rajeev Jha, Simon E. Brill, Ameet Bakhai, Alison Evans, Farhat Gilani, Keith Gomez

**Affiliations:** 1 Katharine Dormandy Haemophilia and Thrombosis Centre, Royal Free London NHS Foundation Trust, London NW3 2QG, UK; 2 Cancer Institute, University College London, London WC1E 6DD, UK; 3 Department of Intensive Care and Anaesthesia, Royal Free London NHS Foundation Trust, London NW3 2QG, UK; 4 Department of Infectious Diseases, Royal Free London NHS Foundation Trust, London NW3 2QG, UK; 5 Respiratory Medicine, Royal Free London NHS Foundation Trust, London NW1 2BU, UK; 6 UCL Respiratory, University College London, London WC1E 6JF, UK; simon.brill@nhs.net; 7 Discovery and Translational Science Department, Leeds Institute of Cardiovascular and Metabolic Medicine, University of Leeds, Leeds LS2 9JT, UK; 8 Freeline Therapeutics, London SG1 2BP, UK; 9 Aberdeen Cardiovascular & Diabetes Centre, School of Medicine, Medical Sciences & Nutrition, Institute of Medical Sciences, University of Aberdeen, Aberdeen AB25 2ZD, UK; 10 Department of Clinical Pharmacy, College of Pharmacy University of Michigan, Ann Arbor, MI 48109, USA; 11 Division of Pulmonary and Critical Care Medicine, School of Medicine, University of Michigan, Ann Arbor, MI 48109, USA; 12 Department of Statistical Science, University College London, London WC1E 6BT, UK; 13 WStats Limited, Winchester SO23 8GH, UK; 14 Haemophilia & Thrombosis Laboratory (Health Services Laboratories), Royal Free Hospital, London WC1H 9AX, UK; 15 Department of Hepatology, Royal Free London NHS Foundation Trust, London NW3 2QG, UK; 16 Clinical Trials Pharmacy, Royal Free London NHS Foundation Trust, London NW3 2QG, UK; 17 Department of Anaesthesia, Birmingham Children’s Hospital, Birmingham B4 6NH, UK; 18 Department of Anaesthesia, Faculty of Medicine, Beni-Suef University Hospital, Beni-Suef University, Beni-Suef 2721562, Egypt; 19 NIHR Biomedical Research Centre Oxford, Oxford University Hospitals NHS Trust, University of Oxford, Oxford OX3 9DU, UK; 20 Bloomsbury Institute for Intensive Care Medicine, Department of Experimental and Translational Medicine, University College London, London WC1E 6JF, UK; 21 Discipline of Acute Care Medicine, School of Medicine, The University of Adelaide, Adelaide, SA 5005, Australia; 22 Department of Respiratory and Critical Care Medicine, Chelsea & Westminster Hospital, London SW10 9NH, UK; 23 Department of Adult Intensive Care, Royal Brompton Hospital, London SW3 6NP, UK; 24 Department of Surgery and Cancer, Imperial College London, London SW7 2AZ, UK; 25 Department of Cardiology, Royal Free London NHS Foundation Trust, London NW3 2PS, UK; 26 University College London (UCL)/University College London Hospitals NHS Trust (UCLH) Joint Research Office, London WC1E 6BT, UK; a.j.evans@ucl.ac.uk (A.E.);

**Keywords:** acute respiratory illness, critical care, recombinant tissue plasminogen activator, nebulization, fibrinolytics, COVID-19 pandemic, inhaled medication, targeted therapy

## Abstract

Acute lung injury in COVID-19 results in diffuse alveolar damage with disruption of the alveolar-capillary barrier, coagulation activation, alveolar fibrin deposition and pulmonary capillary thrombi. Nebulized recombinant tissue plasminogen activator (rt-PA) has the potential to facilitate localized thrombolysis in the alveolar compartment and improve oxygenation. In this proof-of-concept safety study, adults with COVID-19-induced respiratory failure and a <300 mmHg PaO_2_/FiO_2_ (P/F) ratio requiring invasive mechanical ventilation (IMV) or non-invasive respiratory support (NIRS) received nebulized rt-PA in two cohorts (C1 and C2), alongside standard of care, between 23 April–30 July 2020 and 21 January–19 February 2021, respectively. Matched historical controls (MHC; n = 18) were used in C1 to explore efficacy. Safety co-primary endpoints were treatment-related bleeds and <1.0–1.5 g/L fibrinogen reduction. A variable dosing strategy with clinical efficacy endpoint and minimal safety concerns was determined in C1 for use in C2; patients were stratified by ventilation type to receive 40–60 mg rt-PA daily for ≤14 days. Nine patients in C1 (IMV, 6/9; NIRS, 3/9) and 26 in C2 (IMV, 12/26; NIRS, 14/26) received nebulized rt-PA for a mean (SD) of 6.7 (4.6) and 9.1(4.6) days, respectively. Four bleeds (one severe, three mild) in three patients were considered treatment related. There were no significant fibrinogen reductions. Greater improvements in mean P/F ratio from baseline to study end were observed in C1 compared with MHC (C1; 154 to 299 vs. MHC; 154 to 212). In C2, there was no difference in the baseline P/F ratio of NIRS and IMV patients. However, a larger improvement in the P/F ratio occurred in NIRS patients (NIRS; 126 to 240 vs. IMV; 120 to 188) and fewer treatment days were required (NIRS; 7.86 vs. IMV; 10.5). Nebulized rt-PA appears to be well-tolerated, with a trend towards improved oxygenation, particularly in the NIRS group. Randomized clinical trials are required to demonstrate the clinical effect significance and magnitude.

## 1. Introduction

SARS-CoV-2 (COVID-19)-induced respiratory failure is the leading cause of COVID-19 mortality [1,2]. The respiratory failure in severe COVID-19 starts as acute lung injury (ALI) progressing to acute respiratory distress syndrome (ARDS), multiorgan failure and death [3]. ALI and ARDS are characterized by extravascular fibrin deposition in the alveolar compartment due to alveolar cell damage and disruption of the alveolar-capillary barrier [4]. This fibrin deposition is essential for maintaining the temporary integrity of the alveolar-capillary barrier and its subsequent repair [5]. SARS-CoV-2 infection is also characterized by cytokine storm or cytokine response syndrome (CRS), with pronounced elevations of pro-inflammatory cytokines [6]. The relative contribution of the viral cytotoxicity and CRS to the diffuse alveolar damage is not well understood. However, the reduction in mortality with immunomodulation, including steroids and JAK-2 inhibitors, confirms the significant contribution of inflammation to mortality [7,8].

The fibrin deposits in ALI in COVID-19 and other conditions with ARDS are associated with cellular debris and infiltration of inflammatory cells [9]. This is facilitated by increased tissue factor expression and coagulation activation, with suppression of fibrinolysis due to a rise in plasminogen activator inhibitor-1 (PAI-1) activity [10,11]. This disruption to the fibrinolytic system and the subsequent enhanced fibrin deposition in the lungs appears to be a major pathophysiological driver of severe lung disease [5].

Fibrinolytic agents including tissue plasminogen activator (tPA), urokinase-type plasminogen activator (uPA), plasminogen and plasmin are being explored to counteract PAI-1-induced dysregulation of the fibrinolytic system [5]. Nebulized recombinant tPA (rt-PA) enhanced the bronchoalveolar fibrinolytic system in rat models of direct and indirect ALI, as reflected by a significant reduction of PAI–1 activity levels in bronchoalveolar lavage fluid, and a consequent increase in plasminogen activator activity (PAA) [12]. A meta-analysis of 22 studies deemed fibrinolytic therapy an effective therapeutic approach for ALI in pre-clinical models due to the observed improvements in lung injury, oxygenation, local neutrophil infiltration, and mortality following treatment [13]. Three cases of off-label use of tPA administered intravenously in patients with COVID-19-related acute respiratory distress syndrome (ARDS) resulted in a temporary improvement in respiratory status, with one durable response [14]. Moreover, intravenous tPA with immediate therapeutic heparin anticoagulation improved oxygenation in a Phase 2 clinical trial among patients with severe COVID-19 respiratory failure [15].

Fibrinolytic agents are usually administered intravenously, resulting in a systemic increase in fibrinolysis [12,16]. Fibrinolytic therapy, therefore, poses a risk of potentially fatal bleeds. In fact, up to 7% of patients exposed to fibrinolytic agents require blood transfusions, and up to 1% die as a consequence of bleeds [17]. Considering that coagulopathy in ALI involves both alveolar and vascular compartments, local administration via nebulization is an attractive option with potentially higher efficacy and reduced bleeding risk [12,16]. In direct and indirect ALI models, nebulization of rt-PA or anti-PAI-1 demonstrated lung-protective effects via promotion of fibrinolysis [12]. Moreover, inhalation of plasminogen improved lung lesion condition and oxygen saturation in patients with clinically moderate to severe COVID-19 [18].

Essentially, COVID-19 is a multi-system disorder with alveolar and pulmonary vascular inflammatory thrombosis that might benefit from combination therapies addressing both inflammation and intravascular thrombosis or alveolar fibrin deposits to improve outcomes [19]. We hypothesized that nebulized rt-PA through local thrombolysis, alongside standard of care, would improve oxygenation without the excess bleeding risk seen with systemic thrombolysis. This proof-of-concept pilot study aimed to test the safety of nebulized rt-PA and investigate its clinical efficacy in patients hospitalized with COVID-19 respiratory failure that required respiratory support.

## 2. Materials and Methods

### 2.1. Study Design and Participants

This study (clinicaltrials.gov identifier: NCT04356833) was approved by the National Research Ethics Committee (REC) and Medicines and Healthcare Products Regulatory Agency. Health Research Authority (HRL) approval was granted on 17 April 2020 (REC reference: 20/SC/0187). Procedures followed were in accordance with the ethical standards of the International Council for Harmonisation of Technical Requirements for Registration of Pharmaceuticals for Human Use Good Clinical Practice (ICH GCP) guidelines and with the Declaration of Helsinki of 2013.

Written informed consent was obtained from patients. When a patient could not give written informed consent due to intubation and sedation, the study was discussed with the patient’s next of kin, and consent was obtained from an independent professional representative, typically another intensive care consultant not involved in the direct care of the patient or involved in the study. Patients consented at the first opportunity after regaining consciousness and consent could be withdrawn at any time. Supplementary Methods contains the informed consent procedure.

Recruitment for Cohort one (C1) occurred from 23 April to 30 July 2020, during the first COVID-19 surge. Sequential recruitment to the standard of care (SOC) arm originally planned for was not feasible as there were very few COVID-19 admissions to the center after the first COVID-19 surge had subsided by August 2020. Due to low recruitment numbers, the protocol was amended following discussions within the Trial Management Group (TMG) and Independent Data Monitoring Committee (IDMC). This allowed for the recruitment of matched historical controls (MHC) retrospectively for comparison with C1 on 15 October 2020. Recruitment for Cohort two (C2) occurred between 21 January and 19 February 2021 and all patients were assigned to receive rt-PA with SOC to accrue safety data. It is to be noted that SOC itself continued to rapidly evolve through the pandemic with the incorporation of new therapies becoming part of SOC, and our comparison between groups reflects SOC of the time in all study groups (C1, MHC and C2).

Further recruitment details are provided in the Supplementary Methods. After enrolment or the first dose of nebulized rt-PA, patients were followed until the end of the study (EOS). EOS was day 28 or earlier in the event of death or discharge. Day one for MHC was when patients met the inclusion criteria.

Inclusion criteria in the treatment arm for both cohorts included COVID-19 diagnosis (confirmed by polymerase chain reaction [PCR] or radiologically [C1, n = 0; C2, n/N = 3/26]); ≥16 years of age; and acute COVID-19 respiratory failure determined by a PaO_2_/FiO_2_ [P/F] ratio of <300 mmHg [20]) that required respiratory support (including invasive mechanical ventilation). A P/F ratio of <300 mmHg was selected to ensure that all severities of respiratory failure from ALI (≤300 mmHg) to ARDs (≤200 mmHg) were included [21]. There was also a recognition that avoiding mechanical ventilation where possible would result in the best possible outcomes. For consistency and anticipating a relatively small recruitment number (given the high number of COVID-related studies at the time) and perceived much poorer outcomes from invasive mechanical ventilation (IMV), the respiratory support was stratified into two broad categories: IMV via an endotracheal tube and non-invasive respiratory support (NIRS) for all other forms of respiratory support. NIRS included non-invasive ventilation (NIV), continuous positive airway pressure (CPAP), high flow nasal oxygen (HFNO) or conventional oxygen therapy (venturi and non-breathing masks). This categorization was to capture a broad range of patients representative of COVID-19 at the time of the study. The type of respiratory support was determined by the clinical team, but the patients had to have a P/F ratio of <300 at study entry.

In IMV patients, the P/F ratio was calculated with the arterial partial pressure of oxygen (PaO_2_, P) and fraction of inspired oxygen therapy (FiO_2_, F) (Table S1) [22]. In NIRS patients, arterial blood gas analysis was often not performed, and PaO_2_ was imputed by non-linear calculation from oxygen saturation on pulse oximetry (SpO_2_), with FiO_2_ calculated from tables based on oxygen flow and device used (Table S2).

The main exclusion criteria for both cohorts were pregnancy, known allergies to rt-PA or excipients of rt-PA, patients not being actively treated or not considered suitable by the investigator, and fibrinogen levels of ≤2.0 g/L or <1.5 g/L in C1 and C2 at screening, respectively.

There was no restriction on the use of any intervention except participation in another clinical trial of a novel Investigational Medicinal Product. Participation in a recovery study was not an exclusion criterion; nor was concomitant use of anticoagulation or antiplatelet therapy, as the diffusion into alveolar space was considered to be minimal to non-existent. The Supplementary Methods provides additional exclusion criteria for C1.

### 2.2. Study Drug and Dosing

Alteplase, rt-PA (Actilyse^®^, Boehringer Ingelheim, Ingelheim am Rhein, Germany) was reconstituted with 5 mL sterile water (2 mg/mL) and administered using an Aerogen^®^ nebulizer. Supplementary Methods provides details of rt-PA administration.

**Figure 1:**
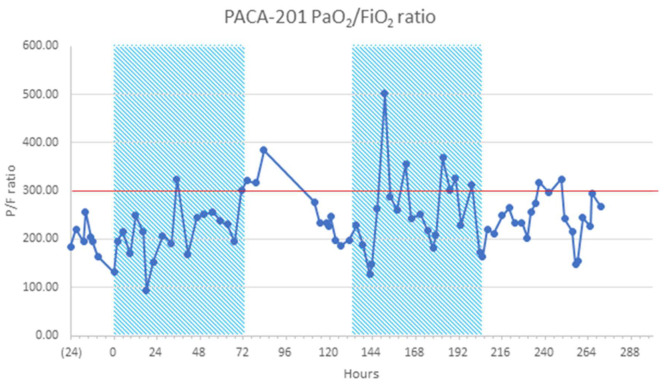
Cohort 1 sample mean PaO_2_/FiO_2_ (P/F) ratio over time of relapsed patient on NIRS (HFNO) requiring two blocks of treatment. Red line indicates severe acute COVID-19 respiratory failure determined by a PaO_2_/FiO_2_ [P/F] ratio of <300 mmHg [20]). HFNO, high-flow nasal oxygen; NIRS, non-invasive respiratory support; P/F, PaO_2_/FiO_2_.

The initial dosing regimen in C1 was 10 mg every 6 h for 72 h. Recruitment was staggered to ensure patient safety and details are provided in the Supplementary Methods. Dosing was amended after observing significant desaturation in patient three, 36 h after the last dose of the initial three-day block of rt-PA was administered. Desaturation was considered significant if the P/F ratio dropped to <300 mmHg. The patient received a second three-day block of rt-PA (Figure 1). This led to a protocol amendment with dosing to take place for a minimum of five days, and a maximum of 14 days. The rationale was underpinned by the fact that several factors impact the sensitivity of the alveolar fibrin deposits to tPA effect, including volume of the clot, duration of the clot, amount of plasminogen available for conversion to plasmin and inhibitors of tPA inhibitors. This resulted in a move from a fixed treatment regimen to an endpoint-driven treatment regimen; treatment was discontinued if blood fibrinogen levels fell to <1.5 g/L (potential toxicity due to systemic absorption) or patients no longer required oxygen (resolution of the interalveolar clot burden). Treatment could be restarted within five days from the last dose of treatment if there was a recurrence of COVID-19-induced respiratory symptoms or a worsening of P/F ratio considered related to treatment discontinuation. The frequency of dosing was determined by previous protocols used in plastic bronchitis [23]. Previous pre-clinical studies demonstrated around 50% deposition of the drug with an Aerogen^®^ nebulizer [24,25]. Studies in mice suggest that the clearance rate of intratracheal administered rt-PA is around 4 to 6 h [26].

## 3. Details of the C2 Treatment Regimen

C2 on IMV received 20 mg rt-PA every eight hours (60 mg daily) for a maximum of 14 treatment days. For patients on NIRS, a loading dose of 20 mg every eight hours was administered for the first two days (60 mg daily) followed by 20 mg every 12 h (twice daily; 40 mg total) for a total of 14 days. Patients on IMV were given a higher dose to account for wastage in the circuit. If patients deteriorated and required IMV, they could receive a higher treatment dose. Treatment was discontinued if blood fibrinogen levels fell to <1.0 g/L or if the patient maintained normal SpO_2_ on room air for 48 h.

### 3.1. Study Endpoints

Primary endpoints to assess safety were (1) the incidence and severity of major bleeding events directly attributable to the study drug; (2) decrease in fibrinogen levels to <1.0 gm/L (in C1, the threshold was 1.5 gm/L) during treatment period and 48 h after the last dose of treatment; and (3) number and nature of serious adverse events causally related to the treatment. For endpoint (2), a lower threshold was chosen in C2 as there was no evidence of systemic absorption. Patients were reviewed daily for bleeding events, use of anticoagulation, intensity, and antiplatelet drugs. Safety blood tests included a daily coagulation screen with fibrinogen. Treatment was stopped for any major bleed and if the fibrinogen level dropped to <1–1.5 gm/L. All bleeding events were categorized as adverse events (AE) of special interest and evaluated for severity (mild, moderate and severe) and causality; the International Society of Haemostasis and Thrombosis (ISTH) Scientific and Standardisation Committee definition of major bleeding events in patients on anti-hemostatic medications was used to grade severity (Table S3) [27]. A bleeding event was evaluated for relatedness if it occurred within 30 h of the last rt-PA dose. This timeframe was determined based on the estimated 4-to-6-h clearance rate of rt-PA via intratracheal administration, based on pre-clinical data [26]. A conservative 6-h clearance rate estimate was assumed in this study and, therefore, bleeding events were evaluated for relatedness if they occurred within 5 clearance rates (30 h) of rt-PA administration.

The secondary endpoint of efficacy was determined as the change in P/F ratio from baseline (BL), which was assessed daily during treatment, at treatment cessation, and at three- and five-days post-treatment cessation. Other secondary endpoints included changes in clinical status assessed by a 7-point World Health Organization (WHO) ordinal scale until EOS (Table S4), the outcome (discharge, in-patient or death) at EOS, changes in lung compliance (defined as tidal volume/peak inspiratory pressure from BL and absolute values), Sequential Organ Failure Assessment (SOFA) during treatment and through five days after the end of treatment, number of oxygen-, ventilator- and intensive care-free days at EOS, and the number of new oxygen or ventilation requirements before EOS.

### 3.2. Biomarkers of Fibrinolysis

Blood samples were taken for exploratory assessment of potential biomarkers to investigate systemic absorption of tPA. These included, but were not restricted to, plasminogen, alpha-2 antiplasmin (α2AP), tissue plasminogen activator (t-PA), PAI-1 and a range of inflammatory cytokines and coagulation proteins. All other monitoring was done as per routine SOC.

### 3.3. Statistical Analysis

Since the study was conducted early in the pandemic, the planned recruitment numbers were based on feasibility and planned recruitment rate rather than statistical considerations. An Independent Data Monitoring Committee was established to provide oversight of the conduct of the study. This was particularly in relation to the causality of bleeding events, dose escalation strategies and to provide recommendations on the continuation of the study.

Descriptive statistics were used for all AEs, including bleeding events of special interest. In C1, the efficacy analysis compared P/F ratios between the rt-PA group and MHC at the EOS, adjusting for the BL P/F ratio, using a linear regression model. A sensitivity analysis was performed, fitting a similar model that controlled for the length of follow-up, as well as the BL P/F ratio. In a further sensitivity analysis, a mixed effects linear regression model was used to compare groups over time and account for the clustering of ratios within patients using a random effect. The model incorporated all P/F ratio measurements over time, with treatment allocation, time, and BL P/F ratio as fixed effects, together with a random slope for time and a random effect at the patient level.

Analyses of C1 and C2 were undertaken separately. C2 analysis was limited to descriptive statistics. Continuous variables were summarized using a number of observations, mean, standard deviation (SD), median, interquartile range (IQR) and minimum and maximum values. Categorical data were summarized using a number of observations and percentages. Further exploratory and post hoc analyses were conducted, and details of all statistical analyses are provided in Supplementary Methods.

## 4. Results

### 4.1. Cohort 1

In total, 27 patients enrolled in Cohort 1 (Figure S2a); nine patients received nebulized rt-PA with SOC and 18 patients were recruited as MHC receiving SOC only. In the rt-PA group, six (66.6%) patients received IMV, and three (33.3%) patients received NIRS, none of whom progressed to IMV. Patient characteristics of C1 are shown in Table 1.

Seven bleeding events occurred in four of the nine patients during rt-PA treatment (Table 2 and Table S6). These events were reported as AE of special interest and categorized as five mild and two moderate; all resolved completely. All bleeds and AEs were deemed unrelated to rt-PA. The MHC group were not reviewed for bleeding events. In addition, there were no measured decreases in plasma fibrinogen levels (<1.5 g/L) during the treatment period and 48 h after the last dose of rt-PA, nor was there any suggestion of increases in tPA-PAI-1 and plasmin-α2-antiplasmin complexes.

The P/F ratio improved during the 28-day study period in the rt-PA and MHC groups (Table 3). One patient that improved to a P/F ratio > 400 on oxygen supplementation by nasal cannula deteriorated 24 to 36 h after the 12th and final dose of rt-PA (Figure 1). This patient was not a candidate for IMV because of previous bronchiectasis; instead, they were treated twice with rt-PA. This observation prompted a change in the dosing schedule for the remaining three patients in C1.

A sensitivity analysis using a linear mixed effects model showed a higher mean P/F ratio in the rt-PA group compared to the MHC group, with an estimated mean difference of 50.6 (95% confidence interval [CI], 6.7–94.4).

Among the rt-PA group, at the EOS, three (33.3%) patients were discharged before Day 28, five (55.6%) remained as in-patients and one patient (11.1%) had died. In the MHC group, six (33.3%) patients had been discharged before Day 28, two (11.1%) were in-patients and ten (55.6%) had died. Patients in the rt-PA group (n = 9) received treatment for a mean (SD) duration of 6.7 (4.6) days (Table 4 and Table S5).

### 4.2. Cohort 2

Twenty-six patients were enrolled on the second cohort, and all received rt-PA (Figure S2b). At the time of screening, 12 (46.2%) were on IMV and 14 (53.9%) were on NIRS. Of the latter, four required IMV for variable periods. Additional patient characteristics for C2 are shown in Table 1.

Among the 26 patients, there were 25 bleeding events (Table 2 and Table S6); seventeen were in the IMV group, and eight were in the NIRS group. These events were reported as AE of special interest and categorized as 18 mild, five moderate, and two severe. Of these, four bleeding events in three patients were considered possibly related to rt-PA treatment, with one being categorized as a severe AE and the other three as mild (Table 2). No patients experienced fibrinogen levels <1.0 g/L at any time during the study. One patient had a fibrinogen value of 1.0 g/L two and three days after the initiation of rt-PA treatment, which prompted withholding a dose of rt-PA.

In the IMV group, the mean (SD) P/F ratio was 120 (28) the day before the first dose of rt-PA, and a small increase was seen for most patients by their last day of treatment, with a mean increase from BL (SD) of 48 (126) (Table 2 and Figure S4). In patients on NIRS, the mean (SD) P/F ratio was 126 (42) the day before the first dose of rt-PA, and an increase was seen for most patients by their last day of treatment, with a mean change (SD) of 114 (92).

The EOS outcomes (28d) for patients on IMV and NIRS, respectively, were as follows: 33.3% and 14.3% remained as inpatients, 25% and 64.3% had been discharged and 41.7% and 21.4% died. The total mean (SD) treatment duration for C1 (n = 26) was 9.1 (4.6) days. Patients on IMV (n = 12) and NIRS (n = 14) received rt-PA for a mean (SD) of 10.5 (4.2) and 7.9 (4.6) days, respectively (Table 4 and Table S5).

#### 4.2.1. 7-Point World Health Organization (WHO) Scale

**Figure 2:**
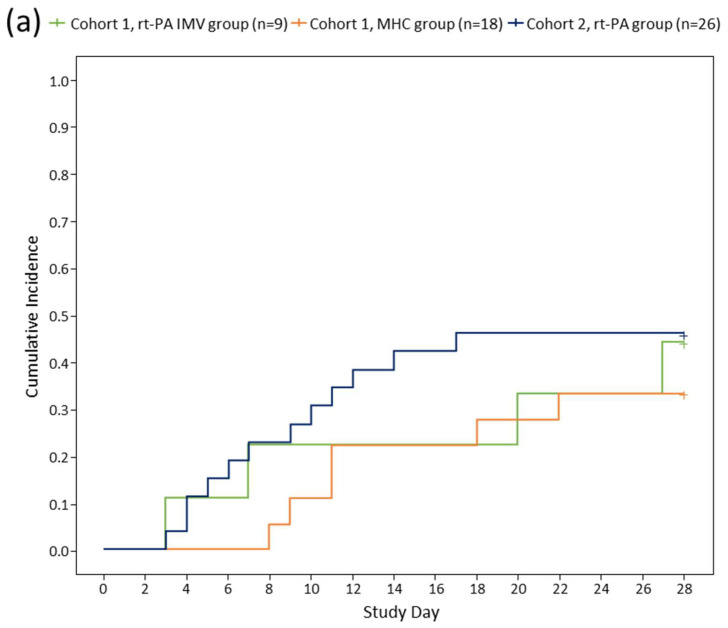

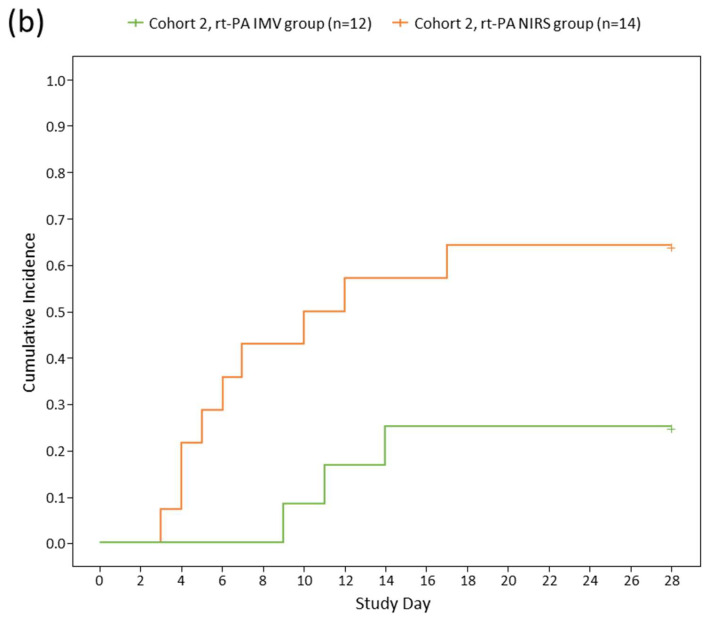
Time to recovery among Cohorts 1 and 2 (**a**); IMV/NIRS subgroups in Cohort 2 (**b**). The graph shows time to recovery, the cumulative incidence of recovery among Cohort 1 and Cohort 2 (**a**) and IMV and NIRS subgroups in Cohort 2 (**b**) (post hoc exploratory results). Time to recovery was defined as the time to achieve a 7-point WHO ordinal score of 1 or 2, or discharge. A breakdown of the WHO ordinal score is provided in Table S4. Data for patients who did not recover and data for patients who died were censored on Day 28. IMV, invasive mechanical ventilation; NIRS, non-invasive respiratory support; rt-PA, recombinant tissue plasminogen activator; MHC, matched historical control.

To explore the treatment effect, a post hoc exploration of the data was conducted to describe the time to recovery from COVID-19 for each patient in the study using the WHO’s minimal common outcome measure set for COVID-19. Recovery was defined as achieving an absolute WHO ordinal score of 1 or 2, or discharge [28]. Data for patients who did not recover or died were censored on Day 28. The exploration of the data aligns with the published literature [29]. The cumulative incidences of recovery during the 28-day study period are shown in Figure 2. In C1, patients on rt-PA had a more rapid recovery compared with MHC patients. In C2, NIRS patients recovered more rapidly than IMV patients. This is likely due to patients on NIRS having lower initial WHO scores, so less recovery was required to achieve a score of 1 or 2 compared with patients on IMV who had higher initial WHO scores (Table S4; Figure 2).

#### 4.2.2. Assessment of Fibrinolysis Biomarkers

The activity of plasminogen, α2AP, PAI-1 antigen (Ag), t-PA Ag and t-PA/PAI complex during rt-PA treatment is presented in the Supplemental Results (Figure S5). There were no significant changes or obvious patterns induced by rt-PA treatment.

## 5. Discussion

This proof-of-concept study is the first clinical trial investigating the use of nebulized rt-PA in patients with COVID-19-induced respiratory failure. Previous reports were limited to intravenous administration of tPA in patients with COVID-19-related respiratory failure [14,15]. Nebulized rt-PA was not associated with any severe excess bleeding and showed an improvement in the P/F ratio among patients with a range of respiratory dysfunction severity. Importantly, the study established a dosing regimen of nebulized rt-PA that was feasible with a tolerable safety profile.

For EOS clinical outcomes, in C1, only one patient (11.0%) receiving rt-PA died during the 28-day study period compared with ten (55.6%) in the MHC group. In C2, five (41.7%) and three (21.4%) patients on IMV and NIRS, respectively, died during the study period. While these findings and an improvement in the P/F ratio were found in the C1 cohort compared with MHC, given the small sample size, they should be viewed as hypothesis-generating and proof-of-concept to support the rationale for a larger, randomized trial.

Alteplase requires plasminogen for its mechanism of action, and therefore, significant bleeding is unlikely due to the low availability of plasminogen. Indeed, the administration of nebulized rt-PA did not appear to induce an increase in systemic markers of fibrinolysis. No patients experienced pulmonary hemorrhage or had clinically significant decreases in systemic fibrinogen. Only a small number of bleeding events and no SAEs of special interest were attributable to rt-PA. In all patients with significant bleeding considered related to rt-PA, concurrent therapeutic anticoagulation with low-molecular-weight heparin (LMWH) was in use, which might have contributed to the bleeding risk. Therapeutic anticoagulation increases the risk of bleeding generally, and this has also been demonstrated in the context of COVID-19 [19,30,31,32]. Furthermore, the safety of nebulized rt-PA has been demonstrated in patients with plastic bronchitis, with a range of doses and durations that did not result in bleeding complications [33]. The use of clinical response for early termination of treatment and an upper limit for treatment duration improves the safety profile. Moreover, our post hoc, exploratory analyses of key fibrinolysis pathway inhibitors revealed that rt-PA treatment did not result in increased systemic fibrinolysis, suggesting minimal absorption, potentially contributing to the favourable safety profile.

Given that this study was conducted in unprecedented times during the COVID-19 pandemic, there are several limitations to this study that may have been prevented if the study was conducted in less unpredictable circumstances.

- The nature of the study population meant that a change in a patient’s condition could result in an alteration in ventilation type post-enrolment. Although all patients had a P/F ratio of <300 at enrolment, the P/F ratio for NIRS and IMV was calculated by different methods: for those on NIRS, the P/F ratio was determined by converting SpO_2_ and oxygen flow rate and using imputed values [22,34], whereas the P/F ratio was readily available for those on IMV. Further, the NIRS group was heterogeneous to the device used to improve oxygen concentration;
- The use of MHC for comparison in C1 is a limitation as patients who consented to participate in trials may differ, potentially leading to selection bias; this has been reviewed extensively [35]. The retrospective, non-randomized nature of the control group makes the efficacy comparison between the control and treatment groups exploratory. Additionally, we acknowledge that the use of historical controls in the absence of randomization may introduce confounding bias. However, one of the main study aims was to generate adequate data for a sample size calculation for a future study;
- This study was not blinded due to the practicalities around blinding this type of intervention, especially in the midst of a pandemic. Due to the lack of blinding, there may be potential biases introduced; however, the aim of this study was to investigate safety and not to demonstrate superiority or gold standard comparisons;
- There were differences in the patients enrolled in the C1 and C2 cohorts, with a higher number of bacterial co-infections in C2 patients, most of whom received steroids and interleukin-6 inhibitors. At the time of the study, both cohorts received the SOC, which was rapidly evolving, as demonstrated by the differences in concomitant treatments (Table 4). It is possible that concomitant treatments received by patients may have impacted the study outcomes. These potential cofounding factors should be explored in future randomized studies;
- The duration of illness before enrolment was shorter in C2; this could have impacted the duration of respiratory support. Reactive protocol amendments were required to incorporate learnings associated with the novel administration route;
- Direct administration of drugs into the airways is challenging, particularly in breathless patients despite the perceived advantages. Dosing of inhaled drugs needs to account for the loss in the ventilation circuit, ambient aerosolization and varying disease severity, and conventional drugs tend to have wide therapeutic windows. Protein-based therapeutics typically have narrow therapeutic windows and tend to be expensive. Whilst the delivery of rt-PA with an Aerogen nebulizer has been investigated extensively [23], the following challenges were experienced in NIRS patients, which may impact the feasibility and effectiveness of nebulized rt-PA treatment in real world settings: (1) Difficulty in continuing to support oxygen whilst using the Aerogen nebulizer for drug administration; (2) the loss of the drug through long circuits used for CPAP and HNFO; (3) trapping of the drug in the filters used for CPAP; (4) taste of the drug when a mouthpiece was used for direct inhalation. Administration with mechanical ventilation was easier due to side ports, but the wastage appeared to be high;
- The assessment of bleeding was complicated by concurrent anticoagulant therapy; whilst this was not a confounder for assessing efficacy, it is an important contributor to the determination of the safety of rt-PA. Indeed, the challenges of the assessment of efficacy and bleeding secondary to anticoagulation in COVID-19 have been extensively reviewed [19,30];
- Lastly, as COVID-19 variants evolve and new therapeutic strategies are developed, the role of salvage therapies like nebulized rt-PA needs careful thought.

Despite these limitations, this study serves as a proof-of-concept that nebulized rt-PA delivery to the airways has a favorable safety profile, even in patients receiving therapeutic anticoagulation with LMWH. The magnitude of clinical impact in relation to the duration of oxygen support, duration of ventilation and need for invasive ventilation needs further assessment. Moreover, the use of nebulized rt-PA for COVID-19-induced respiratory failure, and where this therapy fits into the current COVID-19 disease and treatment landscape will need further exploration.

## 6. Conclusions

In this proof-of-concept study, nebulized rt-PA demonstrated favorable safety with no excess bleeding in patients hospitalized with COVID-19-induced respiratory failure. This requires further investigation in randomized studies to understand both the magnitude and significance of benefit. In addition, there is also a need to better understand the bronchopulmonary hemostatic disturbances and if alveolar fibrin is an appropriate target. These results should be utilized as a first step towards more extended research in the field and will provide valuable scientific knowledge and direction to optimize the design of future studies.

## Figures and Tables

**Table 1:** Details of patient characteristics at baseline in Cohort 1 and Cohort 2.

Patient Characteristics		Cohort 1		Cohort 2
		rt-PA Group (n = 9)	MHC Group (n = 18)	rt-PA Groups (n = 26)
Sex, n (%)	Male	4 (44.4)	9 (50.0)	19 (73.1)
Age, years	Mean	65	67	64
Race, n (%)	Asian	3 (33.3)	4 (22.2)	9 (34.6)
	Black	0	0	1 (3.8)
	White Caucasian	6 (66.7)	10 (55.6)	8 (30.8)
	Other	0	0	5 (19.2)
	Not available/not reported	0	4 (22.2)	3 (11.5)
Ventilation type, n (%)	IMV	6 (66.7)	12 (66.7)	12 (46.2)
	NIRS *	3 (33.3)	6 (33.3)	14 (53.9)
Duration of illness before enrolment	Mean	14.5	8.8	13.1
	Median (min./max.)	8.0 (3/63)	7.0 (0/21)	12.5 (4/27)
Comorbidities of interest, n (%)	Chronic lung disorder	3 (33.3)	0	2 (7.7)
	Chronic heart or circulatory disease	4 (44.4)	9 (50.0)	13 (50)
	Gastrointestinal	2 (22.2)	5 (27.8)	3 (11.5)
	Neurological	1 (11.1)	3 (16.7)	2 (7.7)
	Endocrine	3 (33.3)	4 (22.2)	7 (26.9)
	Chronic haematological	0	1 (5.6)	3 (11.5)
	AIDS/HIV	0	0	0
	Diabetes	3 (33.3)	2 (11.1)	10 (38.5)
	Cancer in the last 12 months	3 (33.3)	1 (5.6)	0
	Rheumatological	2 (22.2)	1 (5.6)	9 (34.6)
	Chronic kidney disease	1 (11.1)	2 (11.1)	0
	Obesity	0	0 ^†^	3 (11.5)
	Dementia	0	2 (11.1)	0
	Immunosuppression	2 (22.2)	2 (11.1)	0

* NIRS included non-invasive ventilation (NIV), continuous positive airway pressure (CPAP), high flow nasal oxygen (HFNO) or conventional oxygen therapy (venturi and non-breathing masks); ^†^ Includes 7 unknown. AIDS, acquired immune deficiency syndrome; ECMO, extracorporeal membrane oxygenation; HIV, human immunodeficiency virus; IMV, invasive mechanical ventilation; MHC, matched historical control; NIRS, non-invasive respiratory support; RRT, renal replacement therapy; WHO, World Health Organization.

**Table 2:** Safety data on bleeding events in Cohort 1 and Cohort 2.

Cohort	Type of Bleed	Events (Patients)	AE Categorisation (Events)	Relatedness to rt-PA (Events) *	Outcome (Events)
1	All	7 (4)	–	–	–
	Central venous catheters insertion site	2 (2)	Mild (2)	NR (2)	Resolved (2)
	Gastro-intestinal bleed	1 (1)	Moderate (1)	NR	Resolved
	Blood-stained tracheobronchial secretion	1 (1)	Mild (1)	NR	Resolved
	Tracheostomy site bleed	2 (2)	Mild (1); Moderate (1)	NR (2)	Resolved (2)
	Other	1 (1)	Mild (1)	NR	Resolved
2	All	25 (13)	–	–	–
	Cerebral bleed ^†^	1 (1)	Severe	NR	Not assessable
	Chest-drain relate ^†^	1 (1)	Severe	R	Not assessable
	GI bleed	2 (2)	Moderate (2)	NR (2)	Resolved (2)
	Blood-stained tracheobronchial secretion	14 (8)	Mild (13); Moderate (1)	R (1)	Resolved (13); Not assessable (1)
	Tracheostomy site bleed	1 (1)	Moderate	NR	Resolved
	Epistaxis	3 (1)	Mild (3)	NR (3)	Resolved (3)
	Other	3 (3)	Mild (2); Moderate (1)	R (2) NR (1)	Resolved (2); Not assessable (1)

* A bleed was evaluated for relatedness if it occurred within 30 h of the last rt-PA dose. Bleeds categorized above minor were managed with stopping of anticoagulation followed by cessation of antiplatelet therapy. Supportive treatment was provided as necessary where there was significant blood loss. Patients were scheduled to receive fibrinogen concentrate if the fibrinogen level dropped to <1.0 g/L. ^†^ This patient developed a tension pneumothorax that required chest drains. Initially, treatment was continued, but three days after the insertion of chest drains, due to ongoing bleeding, both anticoagulation and rt-PA were stopped. The patient was receiving therapeutic anticoagulation for bilateral deep vein thrombosis along with aspirin and the fibrinogen decreased to 1 gm/L concurrent with the administration of tocilizumab. This was considered a moderate, possibly related event. The patient subsequently went on to develop a brain bleed five days after stopping therapy, which was considered unrelated to rt-PA. AE, adverse event; GI, gastrointestinal; ISTH, International Society of Haemostasis and Thrombosis; NR, not related; NSB, non-significant bleeds; R, related; rt-PA, recombinant tissue plasminogen activator.

**Table 3:** Summary statistics for the P/F ratio for Cohort 1, stratified by treatment group and the lowest daily P/F ratio for Cohort 2, stratified by ventilation received alongside rt-PA.

		Cohort 1 * (N = 27)		Cohort 2 ^†^ (N = 26)	
	Parameters	rt-PA Group (n = 9)	MHC Group (n = 18)	IMV Group (n = 12)	NIRS Group (n = 14)
Baseline	n	9	18	12	14
	Mean (SD)	154 (53)	149 (72)	120 (28)	126 (42)
	Median (min./max.)	137 (84/263)	131 (63/268)	121 (71/170)	117 (75/203)
Day 3	n	9	13	12	12
	Mean (SD)	187 (77)	128 (35)	123 (43)	148 (90)
	Median (min./max.)	164 (118/351)	123 (67/202)	112 (43/194)	113 (65/319)
Day 7	n	8	9	10	9
	Mean (SD)	239 (90)	151 (90)	137 (78)	183 (83)
	Median (min./max.)	228 (109/390)	118 (52/305)	150 (30/266)	183 (59/281)
Day 14 ^‡^	n	2	4	8 ^‡^	5 ^‡^
	Mean (SD)	227 (83)	209 (49)	155 (104)	248 (89)
	Median (min./max.)	197 (165/350)	221 (142/262)	149 (43/362)	253 (124/362)
Last On-Treatment Day ^§^	n	9	N/A	12	14
	Mean (SD)	218 (73)	N/A	169 (108)	240 (104)
	Median (min./max.)	211 (1114/338)	N/A	149 (53/362)	281 (60/391)
End of Study ^¶^	n	9	18	12	14
	Mean (SD)	299 (102)	212 (118)	188 (128)	239 (111)
	Median (min./max.)	319 (136/433)	189 (9/433)	173 (43/391)	288 (40/362)

* All available P/F ratio values were extracted per day and summarized every 4 h (±2 h). Time 0 is the baseline and a single time point on the previous day was chosen to illustrate the changes over time. ^†^ Up to six P/F ratio values were extracted per day, including the worst P/F ratio over the preceding day; however, the analysis for Cohort 2 includes only the lowest value for the day. ^‡^ Only thirteen patients (IMV, n = 8; NIRS, n = 5) had an observed measure on Day 14 due to patient discharge or death. ^§^ The last value available on treatment regardless of the duration of treatment (death or discharge may have occurred within the 14 days). ^¶^ Last value available regardless of when this measure occurred (discharge or death may have occurred within 28 days). IMV, invasive mechanical ventilation; MHC, matched historical control; N/A, not applicable; NIV, non-invasive respiratory support; P/F, PaO_2_/FiO_2_; SD, standard deviation; rt-PA, recombinant tissue plasminogen activator.

**Table 4:** Secondary endpoints for Cohort 1 and Cohort 2.

Secondary Endpoint		Cohort 1		Cohort 2	
		rt-PA Group (n = 9)	MHC Group (n = 18)	IMV Group (n = 12)	NIRS Group (n = 14)
End of study outcomes (≤28 days), n (%)					
Discharge		3 (33.3)	6 (33.3)	3 (25.0)	9 (64.3)
Inpatient		5 (55.6)	2 (11.1)	4 (33.3)	2 (14.3)
Death		1 (11.1)	10 (55.6)	5 (41.7)	3 (21.4)
End of study clinical outcomes (≤28 days)–exploratory post-hoc analyses					
Number of oxygen-free days (with imputation *)	Mean (SD)	6.1 (9.6)	N/A	4.42 (8.1)	13.43 (11.1)
	Median (min./max.)	0 (0/24)	N/A	0 (0/20)	17.5 (0/26)
Number of ventilator-free days (with imputation *)	Mean (SD)	11.8 (13)	N/A	5.75 (9·9)	21.4 (9.7)
	Median (min./max.)	10 (0/28)	N/A	0 (0/25)	26.5 (0/28)
New oxygen use (relapse)	Patient, n (%)	0	N/A	1 (8.3%)	0
Progression to IMV		NA	NA	NA	4 (24.6)
Duration of treatment					
n		9	18	12	14
Mean (SD)		6.7 (4.6)	N/A	10.5 (4.2)	7.9 (4.6)
Median (min./max.)		5 (3/14)	N/A	12.8 (2.0/13.7)	8.2 (1.7/13.5)
Important concomitant treatments, n (%) ^†^					
Steroids		7 (77.8)	3 (15.8)	12 (100)	14 (100)
Tocilizumab		0	0 (0)	11 (91.7)	12 (85.7)
Remdesivir		4 (44.4)	0 (0)	8 (66.7)	11 (78.6)
1 type of antibiotic		2 (22.2)	7 (36.8)	2 (16.7)	6 (42.9)
2 types of antibiotics		0	2 (10.5)	2 (16.7)	0
≥3 more types of antibiotics		5 (55.6)	2 (10.5)	8 (66.7)	6 (42.9)
Anakinra		1 (22.2)	2 (10.5)	0	0
Anti-platelet		3 (33.3)	5 (26.3)	3 (25)	5 (35.7)
Anticoagulation–highest intensity		9 (100)	17 (89.5)	12 (100)	14 (100)
Therapeutic		6/9	4 (21.1)	7/12	10/14
Intermediate		1/9	2 (10.5)	5/12	2/14
Prophylactic		2/9	11 (57.9)	0	2/14

* Post-hoc calculation where days post-patient discharge are assumed to be days without oxygen or ventilation; ^†^ Exploratory post-hoc analyses. HFNO, high flow nasal oxygen; IMV, invasive mechanical ventilation; MHC, matched historical control; NIV, non-invasive respiratory support; rt-PA, recombinant tissue plasminogen activator; SOFA, Sequential Organ Failure Assessment; SD, standard deviation; WHO, World Health Organization.

## Data Availability

All data generated or analysed during this study are included in this published article and its Supplementary Information files. Anonymous, individual participant data that underline the results reported in this article will be made available to others, along with the study protocol, statistical analysis plans and information sheets at publication, with no end date. The data will be made available on application to the corresponding author, with permissions from the Chief Investigator and study sponsor (p.chowdary@ucl.ac.uk; a.j.evans@ucl.ac.uk). Applications for data sharing will need to explain what the data will be used for. If the application is approved by the authors, the data will be shared. Data request proposals should be directed to the CI, p.chowdary@ucl.ac.uk. To gain access, data requestors will need to sign a data-sharing agreement. The following abstract has previously been published: A pilot, open-label, phase II clinical trial of nebulized recombinant tissue-plasminogen activator in patients with COVID-19 acute respiratory distress syndrome: the PACA trial. Presented at the British Society for Haemostasis and Thrombosis conference. Aberdeen, United Kingdom. 2022. Abstract OC-15.

## Supplementary Materials

The following supporting information can be downloaded at: https://www.mdpi.com/article/10.3390/jcm12185848/s1, Table S1. Look up table for imputed PaO_2_ for a given SpO_2_ based on a non-linear equation. Table S2. Oxygen flow rate calculation (used for calculating FiO_2_ in patients receiving NIRS). Table S3. Assessment of adverse events for severity, causality, seriousness, and expectedness. Table S4. 7-point WHO ordinal score. Table S5. Secondary endpoints for Cohort 1 and Cohort 2. Table S6. Full safety data on bleeding events in Cohort 1 and Cohort 2. Figure S1. Schematic diagram of overall trial design. Figure S2. CONSORT diagram for Cohort 1 (a) and Cohort 2 (b) study. Figure S3. Cohort 1–Mean P/F ratio over time (days since baseline) stratified by trial arm. Figure S4. Cohort 2–Lowest daily P/F ratio over time for IMV and NIRS subgroups. Figure S5. Biomarker activity during rt-PA treatment in Cohort 1, Cohort 2 IMV, and Cohort 2 NIRS.

## References

[1] Lu S., Huang X., Liu R., Lan Y., Lei Y., Zeng F., Tang X., He H. (2022). Comparison of COVID-19 Induced Respiratory Failure and Typical ARDS: Similarities and Differences. Front. Med..

[2] Ruan Q., Yang K., Wang W., Jiang L., Song J. (2020). Clinical predictors of mortality due to COVID-19 based on an analysis of data of 150 patients from Wuhan, China. Intensive Care Med..

[3] Hussain M., Syed S.K., Fatima M., Shaukat S., Saadullah M., Alqahtani A.M., Alqahtani T., Bin Emran T., Alamri A.H., Barkat M.Q. (2021). Acute Respiratory Distress Syndrome and COVID-19: A Literature Review. J. Inflamm. Res..

[4] Matthay M.A., Zemans R.L., Zimmerman G.A., Arabi Y.M., Beitler J.R., Mercat A. (2019). Acute respiratory distress syndrome. Nat. Rev. Dis. Primers.

[5] Idell S. (2003). Coagulation, fibrinolysis, and fibrin deposition in acute lung injury. Crit. Care Med..

[6] Montazersaheb S., Khatibi S.M.H., Hejazi M.S., Tarhriz V., Farjami A., Sorbeni F.G., Farahzadi R., Ghasemnejad T. (2022). COVID-19 infection: An overview on cytokine storm and related interventions. Virol. J..

[7] Horby P., Lim W.S., Emberson J.R., Mafham M., Bell J.L., Linsell L., Staplin N., Brightling C., Ustianowski A., Recovery Collaborative Group (2021). Dexamethasone in Hospitalized Patients with COVID-19. N. Engl. J. Med..

[8] Kalil A.C., Patterson T.F., Mehta A.K., Tomashek K.M., Wolfe C.R., Ghazaryan V., Marconi V.C., Ruiz-Palacios G.M., Hsieh L., Kline S. (2021). Baricitinib plus Remdesivir for Hospitalized Adults with COVID-19. N. Engl. J. Med..

[9] Bellingan G.J. (2002). The pulmonary physician in critical care * 6: The pathogenesis of ALI/ARDS. Thorax.

[10] Idell S., James K.K., Coalson J.J. (1992). Fibrinolytic activity in bronchoalveolar lavage of baboons with diffuse alveolar damage: Trends in two forms of lung injury. Crit. Care Med..

[11] Whyte C.S., Morrow G.B., Mitchell J.L., Chowdary P., Mutch N.J. (2020). Fibrinolytic abnormalities in acute respiratory distress syndrome (ARDS) and versatility of thrombolytic drugs to treat COVID-19. J. Thromb. Haemost..

[12] Hofstra J.J., Cornet A.D., Declerck P.J., Dixon B., Aslami H., Vlaar A.P.J., Roelofs J.J., van der Poll T., Levi M., Schultz M.J. (2013). Nebulized fibrinolytic agents improve pulmonary fibrinolysis but not inflammation in rat models of direct and indirect acute lung injury. PLoS ONE.

[13] Liu C., Ma Y., Su Z., Zhao R., Zhao X., Nie H.-G., Xu P., Zhu L., Zhang M., Li X. (2018). Meta-Analysis of Preclinical Studies of Fibrinolytic Therapy for Acute Lung Injury. Front. Immunol..

[14] Wang J., Hajizadeh N., Moore E.E., McIntyre R.C., Moore P.K., Veress L.A., Yaffe M.B., Moore H.B., Barrett C.D. (2020). Tissue plasminogen activator (tPA) treatment for COVID-19 associated acute respiratory distress syndrome (ARDS): A case series. J. Thromb. Haemost..

[15] Barrett C.D., Moore H.B., Moore E.E., Wang J., Hajizadeh N., Biffl W.L., Lottenberg L., Paterl P.R., Truitt M.S., Mclntyre R.C. (2022). Study of Alteplase for Respiratory Failure in SARS-CoV-2 COVID-19: A Vanguard Multicenter, Rapidly Adaptive, Pragmatic, Randomized Controlled Trial. Chest.

[16] Camprubí-Rimblas M., Tantinyà N., Bringué J., Guillamat-Prats R., Artigas A. (2018). Anticoagulant therapy in acute respiratory distress syndrome. Ann. Transl. Med..

[17] Global Use of Strategies to Open Occluded Coronary Arteries (GUSTO III) Investigators (1997). A comparison of reteplase with alteplase for acute myocardial infarction. N. Engl. J. Med..

[18] Wu Y., Wang T., Guo C., Zhang D., Ge X., Huang Z., Zhou X., Li Y., Peng Q., Li J. (2020). Plasminogen improves lung lesions and hypoxemia in patients with COVID-19. QJM.

[19] Chowdary P. (2022). COVID-19 coagulopathy—What should we treat?. Exp. Physiol..

[20] Ranieri VI T.O., Rubenfeld G.D., Thompson B.T., Ferguson N.D., Caldwell E., Fan E., Camporota L. (2012). Acute respiratory distress syndrome: The Berlin Definition. JAMA.

[21] Bilan N., Dastranji A., Ghalehgolab Behbahani A. (2015). Comparison of the spo_2_/fio_2_ ratio and the pao_2_/fio_2_ ratio in patients with acute lung injury or acute respiratory distress syndrome. J. Cardiovasc. Thorac. Res..

[22] Brown S.M., Duggal A., Hou P.C., Tidswell M., Khan A., Exline M., Park P.K., Schoenfeld D., Liu M., Grissom C.K. (2017). Nonlinear Imputation of PaO_2_/FIO_2_ from SpO_2_/FIO_2_ among Mechanically Ventilated Patients in the ICU: A Prospective, Observational Study. Crit. Care Med..

[23] Dunn J.S., Nayar R., Campos J., Hybertson B.M., Zhou Y., Manning M.C., Repine J.E., Stringer K.A. (2005). Feasibility of tissue plasminogen activator formulated for pulmonary delivery. Pharm. Res..

[24] Labiris N.R., Dolovich M.B. (2003). Pulmonary drug delivery. Part II: The role of inhalant delivery devices and drug formulations in therapeutic effectiveness of aerosolized medications. Br. J. Clin. Pharmacol..

[25] O’Callaghan C., Barry P.W. (1997). The science of nebulised drug delivery. Thorax.

[26] Lackowski N.P., Pitzer J.E., Tobias M., Van Rheen Z., Nayar R., Mosharaff M., Stringer K.A. (2010). Safety of prolonged, repeated administration of a pulmonary formulation of tissue plasminogen activator in mice. Pulm. Pharmacol. Ther..

[27] Schulman S., Kearon C., The Subcommittee on Control of Anticoagulation of the Scientific and Standardization Committee of the International Society on Thrombosis and Haemostasis (2005). Definition of major bleeding in clinical investigations of antihemostatic medicinal products in non-surgical patients. J. Thromb. Haemost..

[28] Marshall J.C., Murthy S., Diaz J., Adhikari N.K., Angus D.C., Arabi Y.M., Baillie K., Buer M., Berry S., Blackwood B. (2020). A minimal common outcome measure set for COVID-19 clinical research. Lancet Infect. Dis..

[29] Beigel J.H., Tomashek K.M., Dodd L.E., Mehta A.K., Zingman B.S., Kalil A.C., Elizabeth Hohmann M.P.H., Chu H.Y., Annie Luetkemeter M.P.H., Kline S. (2020). Remdesivir for the Treatment of Covid-19—Final Report. N. Engl. J. Med..

[30] Connors J.M., Moll M., Levy J.H. (2021). Interpreting recent clinical studies for COVID-19: A continual process with more new data. Anaesth. Crit. Care Pain. Med..

[31] Musoke N., Lo K.B., Albano J., Peterson E., Bhargav R., Gul F., DeJoy R., Salacup G., Pelayo J., Tipparaju P. (2020). Anticoagulation and bleeding risk in patients with COVID-19. Thromb. Res..

[32] Klok F.A., Huisman M.V. (2020). How I assess and manage the risk of bleeding in patients treated for venous thromboembolism. Blood.

[33] Colaneri M., Quarti A., Pozzi M., Gasparini S., Carloni I., de Benedictis F.M. (2014). Management of plastic bronchitis with nebulized tissue plasminogen activator: Another brick in the wall. Ital. J. Pediatr..

[34] Brown S.M., Grissom C.K., Moss M., Rice T.W., Schoenfeld D., Hou P.C., Thompson B.T., Brower R.G., NIH/NHLBI PETAL Network Collaborators (2016). Nonlinear Imputation of PaO_2_/FIO_2_ From SpO_2_/FIO_2_ Among Patients with Acute Respiratory Distress Syndrome. Chest.

[35] Arabi Y.M., Cook D.J., Zhou Q., Smith O., Hand L., Turgeon A.F., Matte A., Mehta S., Graham R., Brierley K. (2015). Characteristics and Outcomes of Eligible Nonenrolled Patients in a Mechanical Ventilation Trial of Acute Respiratory Distress Syndrome. Am. J. Respir. Crit. Care Med..

