# Supplementary material for: Nebulized Recombinant Tissue Plasminogen Activator (rt-PA) for Acute COVID-19-Induced Respiratory Failure: An Exploratory Proof-of-Concept Trial
Source: J Clin Med. 2023 Sep 8;12(18):5848. doi: 10.3390/jcm12185848 (PMC10531875; doi:10.3390/jcm12185848)
Supplement: Supplementary file 1 [file jcm-12-05848-s001.zip › jcm-2487171-supplementary.pdf]

Nebulized Recombinant Tissue Plasminogen Activator (rt-PA) for Acute COVID-19-Induced Respiratory Failure: An Exploratory Proof-of-Concept Trial

Supplemental Methods

Informed consent procedure

For patients in the treatment arm, adequate time must be given for consideration by the participant/legal representative before taking part. Consent will ideally be sought at least 24 hours after being given the study documentation. However, given the need to urgently begin treatment in many of these patients, it may not be justifiable to seek consent at least 24 hours after providing the study documentation, as the patient's condition may deteriorate over this time. In these instances, consent may be sought less than 24 hours after providing the study documentation. It must be recorded in the medical notes when the participant information sheet has been given to the participant/legal representative.

The Investigator or designee will explain that participants are under no obligation to enter the trial and that they can withdraw at any time during the trial, without having to give a reason. However, if the patient lacks the capacity to give consent due to the severity of their medical condition (e.g. acute respiratory failure or need for immediate ventilation), then consent may be obtained from a relative acting as the patient's personal legal representative. If there is no personal legal representative available (which due to restrictions of visitors to the hospital is likely) a professional legal representative (independent clinical responsibility for the patient's medical care or another person appointed by the hospital) will be asked to provide consent on behalf of the patient. Further consent will then be sought from a personal legal representative if one becomes available or from the patient if they recover sufficiently. If the patient refuses consent after recovery, further assessments will be stopped."

For matched historical controls, the initial collection of data will be done without patient consent under The Health Service (Control of Patient Information) Regulations 2002 and the notices issued by the secretary of state to healthcare organisations, general practitioners, local authorities, and arm's length bodies to allow sharing of information to support efforts against coronavirus (COVID-19). These notices are currently due to end on 31 March 2021. Consent will not be sought from historical controls because the use of their personal identifiable information is covered by this notice."

Study design

Recruitment was conducted during the two COVID-19 surges in the UK. Cohort 1 (C1) during the first surge between April 2020 and August 2020 and Cohort 2 (C2) during the second surge between January 2021 and February 2021. A matched historical control group (MHC), at a ratio of two MHC patients to every one patient receiving recombinant tissue-Plasminogen Activator (rt-PA), was obtained from patients in the first surge. Patients were obtained under The Health Service (Control of Patient Information) Regulations 2002 and the notices issued by the secretary of state to healthcare relating to COVID-19 which waives patient consent. There was no control arm for C2 as the major aim was to accrue additional safety data and understand the heterogeneity in treatment response.

The recruitment for C1 closed earlier than planned due to the reduction in COVID-19 cases after the first wave of COVID-19 in August 2020 making recruitment difficult. The data from C1 were analysed and then the protocol was amended to include a second cohort (Figure S1). In addition, given the expected decrease in cases during the second wave of COVID-19 in February 2021, an unplanned data cut and analysis of C2 was performed on 1st April 2021.

The MHC were recruited at a ratio of two controls to every individual rt-PA and MHC arm patient (Figure S1). Of the two sites in the organisation, the site with electronic patient records was chosen for MHC. Deceased patients could be considered as MHC; however, as a certain number of observations were required for plotting the PaO2/FiO2 (P/F) ratio over time, patients with less than two days of data were excluded. Other information extracted included date of admission and discharge, number of oxygen days, stay in the critical care unit, ethnicity, age, sex, and status (alive or deceased). Stay in intensive care was used as a proxy for identifying patients requiring invasive ventilation, and patients were matched using a combination of ethnicity and age (+/– two years, up to a maximum of ten years) starting with a smaller window and expanding until suitable patients were identified. Following the identification of MHC, all numerical data was extracted from health records and imported into the study database. Healthcare records were also reviewed for demographic information and confirmation of COVID-19 diagnosis and outcomes. MHC patients were ‘enrolled’ in the study when two sequential P/F ratios were <300.

All patients recruited to the treatment arms were recruited at the Royal Free Hospital in London. All MHC patients were recruited from Barnet hospital (part of the Royal Free London NHS Foundation Trust).

Participants

For patients receiving NIRS, PaO_2 was imputed by using a reference algorithm and similarly, FiO_2 was derived either from the device or from tables based on the device used (Table S2).

Exclusion criteria

Further exclusion criteria for cohort 1 included incurable lung cancer, severe chronic obstructive lung disease, cardiomyopathy, heart failure or impaired contractility <40% left or right ventricular ejection fraction (LVEF/RVEF), being considered inappropriate for critical care (e.g., being considered for palliative care), active bleeding within the preceding 7 days, or were considered not suitable in the opinion of the investigator. Fibrinogen level at screening of <2.0 g/L was also an exclusion in C1; this threshold was reduced to ≤1.5 g/L in C2.

For IMV patients, a P/F of ≤300 was required, and patients must have been intubated for >6 hours.

NIRS patients also had to be in-patients for >6 hours and be actively treated.

Study drug and drug dosing

The Aerogen Solo Nebulizer System is a portable medical device for single-patient use that is intended to aerosolize physician-prescribed solutions for inhalation to patients on and off ventilation or other positive pressure breathing assistance. (Aerogen Solo System Instruction Manual; www.aerogen.com).

Alteplase (Actilyse) solution was prepared for nebulisation using an aseptic technique immediately before dose administration. A vial of Alteplase powder would be reconstituted with sterile water and the resultant solution transferred to the Aerogen Solo solution chamber of the nebuliser system. The setup of the Aerogen nebuliser and the Alteplase doses depended on the patients' ventilation mode: either the invasive mechanical ventilation (IMV) group or the non-invasive ventilation (NIV) group.

In cohort 1, subjects were given a total daily dose of 40 mg of rt-PA, regardless of their mode of ventilation. Individual doses of 10 mg were administered at four time points, every six hours (± 1 hour) within 24 hours. In cohort 2, the dosing schedule was changed, and subjects received differing doses depending on the ventilation type. Subjects in the IMV group received a total daily dose of 60 mg of rt-PA. Individual doses of 20 mg were administered at three time points, every eight hours (± 2 hours) within 24 hours. A maximum of 42 doses in total could be administered in the IMV group. Subjects in the NIRS group initially received a 'loading dose' of 60 mg total daily dose. These were administered at three time points, every eight hours (± 2 hours) for the first six doses (over 48 hours). By day three, subjects received a 40 mg total daily dose, given at two time points every 12 hours (± 4 hours). A maximum of 30 doses in total could be administered in the NIV group.

Subjects in the NIV group who were conscious and with capacity were trained to use the Aerogen Ultra mouthpiece for optimal drug delivery to the lungs. Subjects using the mouthpiece were trained to take in deep breaths to maximise the drug delivery into the lungs. The solution chamber is attached directly to the Aerogen Ultra mouthpiece, which the subject could hold up to their mouths in their hand during the drug administration process. The solution chamber was connected to the Aerogen Pro-X controller, which activated the nebulisation of the solution. Patients receiving oxygen with a **venturi mask** (who were stable at room air oxygen for short periods) could use the mouthpiece to receive the nebulised Alteplase in this manner. Patients needing minimal ventilatory support received oxygen through **high-flow nasal prongs**. For these subjects, the Aerogen facemask could be used to deliver the nebulised Alteplase without disrupting the supplementary oxygen delivery from the nasal prongs. The facemask could be attached to the piece which docked the solution chamber and connected to the nebuliser. The facemask allowed for the administration of the Alteplase, independent of the nasal prongs delivering the required concentration of oxygen to the patient. For the more severe patients, **continuous positive airway pressure (CPAP)** ventilation was given. This required the patient to wear an airtight mask over their mouth and nose to receive oxygen. In this setup, subjects could not use the Aerogen Ultra mouthpiece, and therefore a T-piece had to be inserted between the mask and the ventilation tube, allowing for the docking of the Aerogen Solo solution chamber. Once attached, the subject could receive their required oxygen concentration using the CPAP equipment whilst simultaneously receiving the nebulised Alteplase doses. On average, it took 15–30 minutes to administer a nebulised dose of rt-PA.

Subjects in the intensive care unit (ICU) receiving IMV were unconscious and unable to use the mouthpiece. Therefore, the solution chamber was attached to the IMV apparatus by the subject’s bedside and the Alteplase solution was added. The Alteplase was nebulised and thereby administered using the IMV apparatus, with minimal interference to the patient, their ventilation setup, or the supplementary oxygen delivery.

Details of the C1 treatment regimen

At the onset of the study, a gap of 24 hours was maintained between the treatment of patient one and patient two to assess the safety of the rt-PA. At 24 hours, if patient one had no evidence of major pulmonary bleeding, suggesting exaggerated alveolar fibrinolysis, and had no evidence of a >50% reduction in fibrinogen, suggesting systemic absorption, then patient two was dosed. Both patients were then evaluated for 72 hours. If no major bleeding was noticed and there was no evidence of a >50% reduction in fibrinogen in either patient, then the rest of the cohort was

recruited after the safety data had been reviewed by the trial management group comprised of the investigators. If the safety profile was deemed acceptable, dosing of the third and subsequent patients in the rt-PA group was resumed with no required interval between patients (Figure S1).

Dosing was later amended after observing significant desaturation in patient three, 36 hours after the last dose of the initial three-day block of rt-PA was administered. An urgent safety measure was implemented to allow re-treatment of this patient with a second 3-day block of rt-PA. This was subsequently followed up with a protocol amendment to allow dosing to take place for a minimum of 5 days, and a maximum of 14 days. This resulted in a move from a fixed treatment regimen to an endpoint-driven treatment regimen.

Primary endpoints

Based on the analysis of C1, fewer P/F ratio data points were collected for C2, which allowed more rapid recruitment without compromising safety monitoring. For C2, data for this measure were extracted once a day for each patient. In addition, up to a maximum of six data points that represented the patient's clinical status over the proceeding 24 hours were extracted.

The P/F ratio for patients receiving NIV was calculated by converting SpO_2 into PaO_2 using conversion tables included in the study protocol. FiO_2 was calculated by converting the oxygen flow rate (in L/min) into FiO_2, using tables provided in the study protocol appropriate for the ventilation type. The imputed PaO_2 and FiO_2 were also entered into the Case Report Form (CRF) and database.

Assessments of adverse events

Each adverse event was assessed for severity, causality, seriousness, and expectedness. A full breakout of the categories and definitions is presented in Table S3. The International Society of Haemostasis and Thrombosis (ISTH) classification for bleeding severity was used to evaluate bleed severity.

Secondary endpoints

The breakdown of the 7-point World Health Organisation (WHO) ordinal scale is presented in Table S4.

Exploratory biomarkers of fibrinolysis

Additional samples were taken for exploratory assessment of potential biomarkers including, but not restricted to, plasminogen activator inhibitor-1 (PAI-1), alpha-2 antiplasmin (α2AP), and a range of inflammatory cytokines and coagulation proteins. All other monitoring was done as per routine SOC.

Stopping rules

Treatment was to be discontinued in an individual patient if blood fibrinogen levels fell to <1.5 g/L (C1) or <1.0 g/L (C2) or if the patient maintained normal SpO2 on room air for 48 hours.

The trial may be stopped before completion for the following reasons:

- On the recommendation of the sponsor and Chief Investigator
- A major pulmonary bleeding that is symptomatic or fatal that occurs between the start of treatment and 30 hours after the last dose
- In Cohort 1, a 50% or greater reduction of fibrinogen at 72 hrs (from baseline value at screening) in either of the first two patients after dosing. If a 50% or greater drop in fibrinogen is observed in a patient, it would only be a particular safety concern in that patient if the fibrinogen were to go below 1.5 g/L (hence why this level is stated as a reason to stop dosing in an individual patient). However, if a 50% drop in fibrinogen is observed from baseline values in either of the first two patients it might suggest that the dose is too high overall with secondary systemic absorption. As such stopping the trial will provide the opportunity to discuss within the Trial Management Group and Independent Data Monitoring Committee to decide on the dose going forward. A substantial amendment would be made to the Medicines and Healthcare products Regulatory Agency and Research Ethics Committee
- Any fatal bleeding that occurs between the start of treatment and 30 hours after the last dose
- Symptomatic bleeding in a critical area or organ, such as intracranial, intraspinal, pericardial, pulmonary, or intramuscular with compartment syndrome, that occurs between the start of treatment and 30 hours after the last dose.

Statistical analysis

Following the review of data collection in C1, and the decision to reduce the amount of data collected for C2, two changes to the planned analyses were implemented. Firstly, due to the numerous and repeated efficacy measures available for each patient, the date and time of the first dose of rt-PA were used as the start of treatment. Treatment days were therefore defined by 24-hour increments from this first dose, rather than based on calendar days. Secondly, given the high occurrence of patients discharged or passing away during the study, the data over time is limited. Results were therefore produced for the last on-treatment day and end-of-study. This type of summary is similar in principle to the last observation carried forward, using the last measure before

the last dose of rt-PA, or within 28 days, regardless of when this measure occurred (discharge or death may have occurred). These imputed results are presented in this manuscript.

Post-hoc exploration of the data

- Post-hoc exploration of the data from both cohorts was produced for this manuscript: The time to recovery based on the 7-point WHO ordinal score and biomarkers data were plotted for C1 and C2 (see methods in the main manuscript)
- The number of Oxygen-free days and Ventilator free days were recalculated to consider the number of days post-discharge. For this new calculation, days post-discharge was assumed to be days when the patient did not receive oxygen or ventilation. In addition, for each patient: “New oxygen use (relapse)” was defined as any patient requiring oxygen support after being on room air for a whole day, and “New mechanical ventilation use (deterioration)” was defined as any patient requiring mechanical ventilation after a period of non-mechanical ventilation after receiving r-tPA
- Classes of medications including antiplatelet agents and anticoagulation and intensity of anticoagulation (prophylactic, intermediate, therapeutic) were assigned to all medications by the Principal Investigator (PI) and summarised for treated patients in both cohorts (Table 4 in the main manuscript). The summary includes all medication received by the patient from the first day of dosing, until day 28 and includes medication initiated during admission before the first day of dosing which can impact the outcomes of COVID. Except for antibiotics, for which all instances received post-admission (even after Day 28) are counted. For anticoagulants, the highest type received was also summarized
- Bleeds were assigned a type by the PI (Table 4 in the main manuscript) and comorbidities of interest were summarised (Table 1 in the main manuscript)
- The activity of plasminogen, α2AP, PAI-1 Ag, tPA Ag and rtPA/PAI complex was plotted for each patient, per cohort. Missing data was not imputed but any values reported as <x or > x were imputed by x for these figures

Supplemental Results

Cohort 1 (C1)

Secondary endpoints

Changes in lung compliance

Mean lung compliance over time for both the treatment group and the MHC group fluctuated during the 28-day study period. However, overall lung compliance was higher in the treatment group (Table S5). The loss of patients due to death and discharge had a disproportionate impact on the values due to the small sample size, making the interpretation not meaningful.

Mean daily SOFA score

The mean SOFA score over time for both the treatment group and the MHC group fluctuated during the 28-day study period. However, the overall SOFA scores for the treatment group were lower (Table S5). The loss of patients due to death and discharge had a disproportionate impact on the values due to the small sample size, making the interpretation not meaningful.

Cohort 2 (C2)

Secondary endpoints

Changes in lung compliance

Among the patients receiving IMV (n=12), the mean (SD) lung compliance at the start of treatment was 40.4 (38.0), available for 10 patients. On the last day of treatment, the mean (SD) lung compliance was 37.6 (42.8) (Table S5). The loss of patients due to death and discharge had a disproportionate impact on the values due to the small sample size, making the interpretation not meaningful.

Mean daily Sequential Organ Failure Assessment (SOFA) score

Among all patients, the mean (SD) baseline SOFA score was 3.6 (3.21) (n=25) at the start of treatment. During the treatment period, the SOFA score fluctuated while gradually increasing; on the last on-treatment day a mean (SD) SOFA score of 6.3 (5.30) was reported (Table S5). The loss of patients due to death and discharge had a disproportionate impact on the values due to the small sample size, making the interpretation not meaningful.

Exploratory biomarker assessments

The activity of plasminogen, α2AP, PAI-1 antigen (Ag), t-PA Ag, and t-PA/PAI complex during rt-PA treatment are shown in Figure S5. There was considerable interpatient variability at BL for many of the parameters. Increases in the levels of t-PA Ag were observed in the NIV patients, with a smaller response in the IMV patients (Figure S5d). In some cases, both the IMV and NIRS groups demonstrated large increases in t-PA/PAI complexes compared to others (Figure S5e). A consistent

pattern was not discernible, suggesting potentially minimal absorption, also demonstrated by a lack of decrease in fibrinogen to below 1.0 g/L as mentioned above.

Table S1. Look up table for imputed PaO_2 for a given SpO_2 based on a non-linear equation

Measured SPO_2 (%)	Imputed PaO_2 (mmHg)	Measured SPO_2 (%)	Imputed PaO_2 (mmHg)
100*	167*†	84	49
99*	132*	83	47
98*	104*	82	46
97*	91*	81	45
96	82	80	44
95	76	79	43
94	71	78	42
93	67	77	42
92	64	76	41
91	61	75	40
90	59	74	39
89	57	73	39
88	55	72	38
87	53	71	37
86	51	70	37
85	50		

*Generally considered unreliable on the basis of the sigmoidal shape of the haemoglobin oxygen dissociation curve; † based on SpO_2 99.5%

Table S2. Oxygen flow rate calculation (used for calculating FiO_2 in patients receiving NIRS)

Oxygen delivery devices			
Device		Flow rate (L/min)	% Oxygen delivered
Nasal cannula		1	24
		2	28
		4	36
Venturi value and mask	Blue	2–4	24
	White	4–6	28
	Yellow	8–10	35
	Red	10–12	40
	Green	12–15	60
Non-rebreather mask		15	85

NIRS, non-invasive respiratory support.

Table S3. Assessment of adverse events for severity, causality, seriousness, and expectedness

Category	Definition
**Severity**	
Mild	The AE does not interfere with the participant's daily routine, and does not require intervention; it causes slight discomfort
Moderate	The AE interferes with some aspects of the participant's routine, or requires intervention, but is not damaging to health; it causes moderate discomfort
Severe	The AE results in alteration, discomfort or disability which is damaging to health
**Causality**	
Related	A causal relationship between an IMP/investigational treatment and an AE is at least a reasonable possibility, i.e., the relationship cannot be ruled out
Not related	There is no reasonable possibility of a causal relationship between an IMP/investigational treatment and AE
**Expectedness**	
Expected	An AE which is consistent with the information about the IMP listed in the current approval References Safety Information for the trial
Unexpected	An AE which is not consistent with the information about the IMP listed in the current approval References Safety Information for the trial
**Seriousness**	
Serious AE	Any AE, adverse reaction, or unexpected adverse reaction, respectively, that:<br>• Results in death<br>• Is life-threatening*

	• Requires hospitalisation or prolongation of existing hospitalisation[†]<br>• Results in persistent or significant disability or incapacity or,<br>• Consists of a congenital anomaly or birth defect

* Refers to an event in which the participant was at risk of death at the time of the event; it does not refer to an event which hypothetically might have caused death if it were more severe;

[†] Hospitalisation is defined as an in-patient admission, regardless of the length of stay. Hospitalisation for pre-existing conditions, including elective procedures does not constitute an SAE

IMP, investigational medicinal product; SAE, serious adverse event.

Table S4. 7-point WHO ordinal score

**Descriptor**	**Score**
Limitations of activities	1
Hospitalised, no oxygen therapy	2
Oxygen by mask or nasal prongs	3
Non-invasive ventilation or high-flow oxygen	4
Intubation and mechanical ventilation	5
Ventilation and additional organ support	6
Death	7

WHO, World Health Organization.

Table S5. Secondary endpoints for Cohort 1 and Cohort 2

		Cohort 1		Cohort 2	
**Secondary endpoint**		**rt-PA group** (n=9)	**MHC group** (n=18)	**IMV group** (n=12)	**NIRS group** (n=14)
**End of study outcomes (≤28 days), n (%)**					
Discharge		3 (33.3)	6 (33.3)	3 (25.0)	9 (64.3)
Inpatient		5 (55.6)	2 (11.1)	4 (33.3)	2 (14.3)
Death		1 (11.1)	10 (55.6)	5 (41.7)	3 (21.4)
**End of study clinical outcomes (≤28 days) – exploratory posthoc analyses**					
Number of oxygen-free days (with imputation*)	Mean (SD)	6.1 (9.6)	N/A	4.42 (8.1)	13.43 (11.1)
	Median (min./ max.)	0 (0/24)	N/A	0 (0/20)	17.5 (0/26)
Number of ventilator-free days (with imputation*)	Mean (SD)	11.8 (13)	N/A	5.75 (9·9)	21.4 (9.7)
	Median (min./ max.)	10 (0/28)	N/A	0 (0/25)	26.5 (0/28)
New oxygen use (relapse)	Patient, n (%)	0	N/A	1 (8.3%)	0
New mechanical ventilation use (deterioration)	Patient, n (%)	0	N/A	1 (8.3%)	2 (14.3%)
**Lung compliance**					
Baseline	n	6	N/A	10	N/A
	Mean (SD)	28 (7.3)	N/A	40.4 (38.0)	N/A

	Median (min./max.)	28 (20.0/38.0)	N/A	26.82 (17.4/144.7)	N/A
Last on-treatment day	n	6	N/A	12	N/A
	Mean (SD)	40.4 (22.5)	N/A	37.6 (42.8)	N/A
	Median (min./max.)	32.0 (17.0/132.0)	N/A	20.8 (-8.79/127.8)	N/A
**WHO Ordinal Scale Class at baseline, n (%)**					
1 – limitation of activities		0	0	0	0
2 – hospitalised, no oxygen		0	0	1 (8.3)	1 (7.1)
3 – oxygen by mask or nasal prongs		2 (22.2)	4 (30.8)	7 (58.3)	11 (78.6)
4 – non-invasive ventilation or HFNO		1 (11.1)	3 (23.1)	1 (8.3)	2 (14.3)
5 – Intubation and mechanical ventilation		2 (22.2)	3 (23.1)	1 (8.3)	0
6 – ventilation and additional oxygen support		4 (44.4)	3 (23.1)	2 (16.7)	0
7 – death		0	0	0	0
**SOFA score at baseline**					
n		9	11	11	14
Mean (SD)		6.78 (4.74)	7.09 (3.9)	5.2 (44)	2.4 (0.9)
Median (min./max.)		7.0 (2/15)	8.0 (2/13)	2.0 (2/13)	2.0 (2/5)
**Duration of treatment**					
n		9	18	12	14
Mean (SD)		6.7 (4.6)	N/A	10.5 (4.2)	7.86 (4.6)
Median (min./max.)		5 (3/14)	N/A	12.8 (2.0/13.7)	8.2 (1.7/13.5)
**Important concomitant treatments, n (%)[†]**					
Steroids		7 (77.8)	N/A	12 (100)	14 (100)
Tocilizumab		0	N/A	11 (91.7)	12 (85.7)

Remdesvir	4 (44.4)	N/A	8 (66.7)	11 (78.6)
1 type of antibiotic	2 (22.2)	N/A	2 (16.7)	6 (42.9)
2 types of antibiotics	0	N/A	2 (16.7)	0
≥3 more types of antibiotics	5 (55.6)	N/A	8 (66.7)	6 (42.9)
Anakinra	1 (22.2)	N/A	0	0
Anti-platelet	3 (33.3)	N/A	3 (25)	5 (35.7)
Anticoagulation – highest intensity	9 (100)	N/A	12 (100)	14 (100)
Therapeutic	6/9	N/A	7/12	10/14
Intermediate	1/9	N/A	5/12	2/14
Prophylactic	2/9	N/A	0	2/14
Type of anticoagulant				
Tinzaparin	6 (66.7)	N/A	12 (100)	14 (100)
Enoxaparin	4 (44.4)	N/A	1 (8.3)	0
Rivaroxaban	1 (11.1)	N/A	0	1 (7.1)
Apixaban	1 (11.1)	N/A	0	4 (28.6)
Heparin	0	N/A	1 (8.3)	0

* Post-hoc calculation where days post-patient discharge is assumed to be days without oxygen or ventilation;

† Exploratory posthoc analyses.

HFNO, high flow nasal oxygen; IMV, invasive mechanical ventilation; MHC, matched historical control; NIV, non-invasive respiratory support; rt-PA, recombinant tissue plasminogen activator; SOFA, Sequential Organ Failure Assessment; SD, standard deviation; WHO, World Health Organization.

Table S6. Full safety data on bleeding events in Cohort 1 and Cohort 2

Cohort	Type of bleed	Events (patients)	AE categorisation (events)	ISTH classification (events)	Anticoagulation (events)	Concomitant antiplatelet (events)	Relatedness to rt-PA (events)	Interruption of rt-PA (events)	Outcome (events)
1	All	7 (4)							
	Central venous catheters access the site	2 (2)	Mild (2)	NSB (2)	Therapeutic (2)	Yes (1)	Not Related (2)	None (2)	Resolved (2)
	Gastro-intestinal bleed	1 (1)	Moderate (1)	Major	Therapeutic	Yes	Not Related	None	Resolved
	Blood-stained tracheobronchial secretion	1 (1)	Mild (1)	NSB	Therapeutic	Yes	Not Related	None	Resolved
	Tracheostomy site bleed	2 (2)	Mild (1)<br>Moderate (1)	NSB (1)<br>Minor (1)	Therapeutic (2)	No (2)	Not Related (2)	None (2)	Resolved (2)

	Other	1 (1)	Mild (1)	NSB	Therapeutic	Yes	Not Related	None	Resolved
2	All	25 (13)							
	Cerebral bleed*	1 (1)	Severe	Major	None	No	Not Related	None	Not assessable
	Chest-drain related*	1 (1)	Severe	Major	Intermediate (1)	Yes	Related	Stopped	Not assessable
	GI bleed	2 (2)	Moderate (2)	Major (2)	Therapeutic (2)	Yes (1)<br>No (1)	Not Related (2)	Interrupted (1)<br>None (1)	Resolved (2)
	Blood-stained tracheobronchial secretion	14 (8)	Mild (13)<br>Moderate (1)	Non-significant (11)<br>Minor (3)	Therapeutic (3)<br>Intermediate (11)	No (14)	Related (1)	Interrupted (1)	Resolved (13)<br>Not assessable (1)
	Tracheostomy site bleed	1 (1)	Moderate	Minor	Intermediate (1)	No	Not Related	None	Resolved

	Epistaxis	3 (1)	Mild (3)	NSB (2)<br>Minor (1)	Therapeutic (2)<br>Prophylactic (1)	No (3)	Not Related (3)	None (3)	Resolved (3)
	Other	3 (3)	Mild (2)<br>Moderate (1)	NSB (1)<br>Minor (2)	Therapeutic (2)<br>Intermediate (1)	Yes (2)	Related (2)<br>Not related (1)	Stopped (1)<br>Interrupted (1)<br>None (1)	Resolved (2)<br>Not assessable (1)

* This patient developed a tension pneumothorax that required chest drains. Initially, treatment was continued, but three days after the insertion of chest drains, due to ongoing bleeding both anticoagulation and rt-PA were stopped. The patient was receiving therapeutic anticoagulation for bilateral deep vein thrombosis along with aspirin and their fibrinogen decreased to 1 gm/L concurrent with the administration of tocilizumab. This was considered a moderate, possibly related event. The patient subsequently went on to develop a brain bleed, five days after stopping therapy which was considered unrelated to rt-PA.

AE, adverse event; GI, gastrointestinal; ISTH, International Society of Haemostasis and Thrombosis; NSB, non-significant bleeds; rt-PA, recombinant tissue plasminogen activator.

Figure S1. Schematic diagram of overall trial design

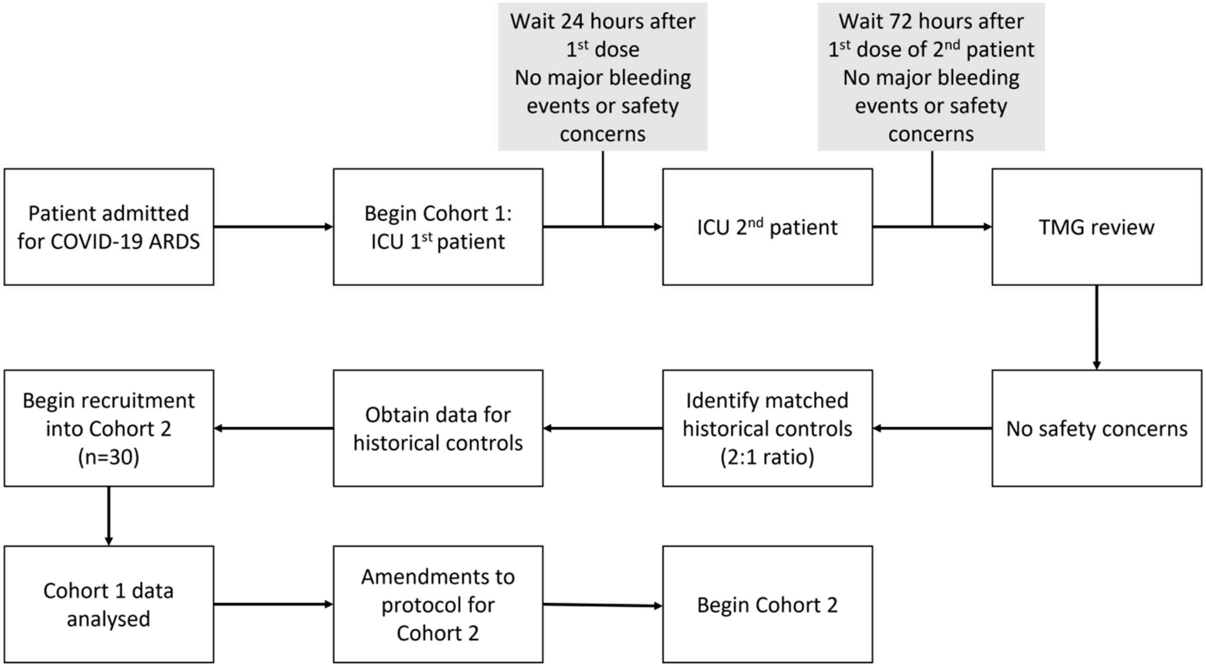

ARDS, acute respiratory distress syndrome; ICU, intensive care unit; TMG, trial management group.

Figure S2. CONSORT diagram for Cohort 1 (a) and Cohort 2 (b) study

a)

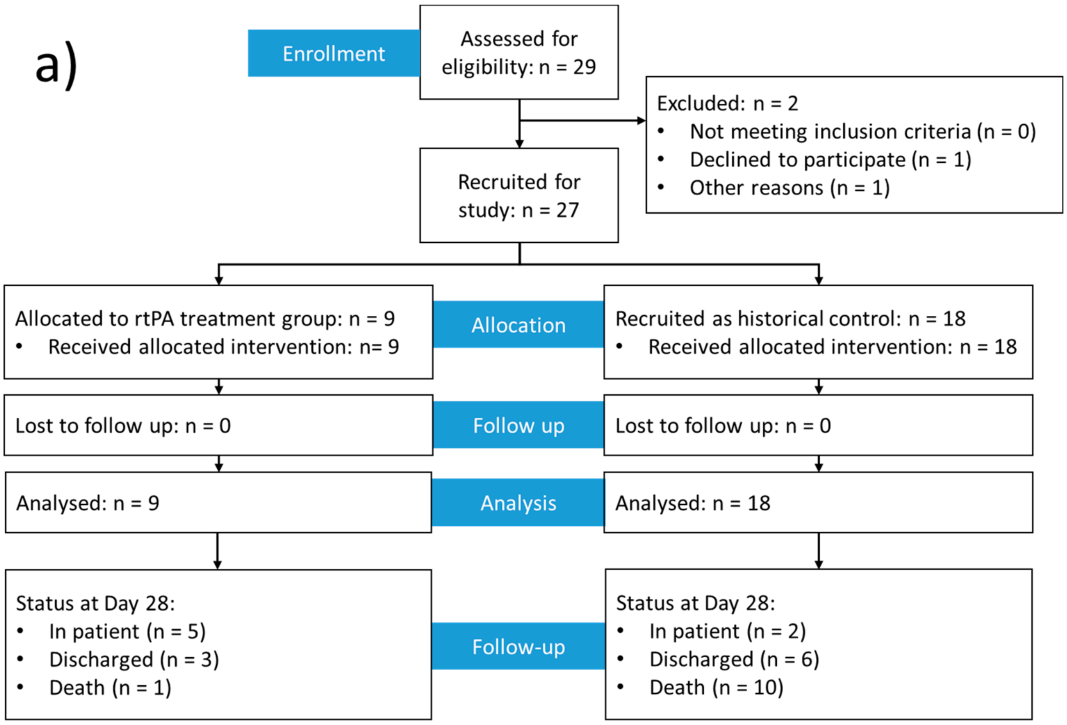

b)

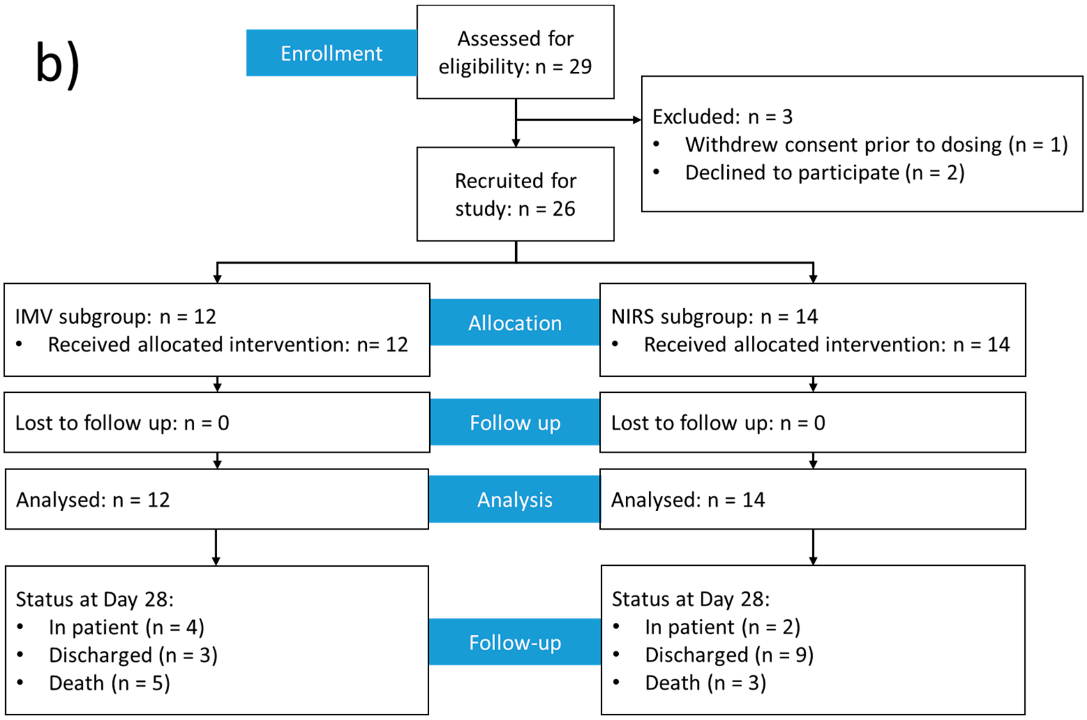

IMV, invasive machinal ventilation; NIRS, non-invasive respiratory support; rt-PA, recombinant tissue plasminogen activator.

Figure S3. Cohort 1 - Mean P/F ratio over time (days since baseline) stratified by trial arm

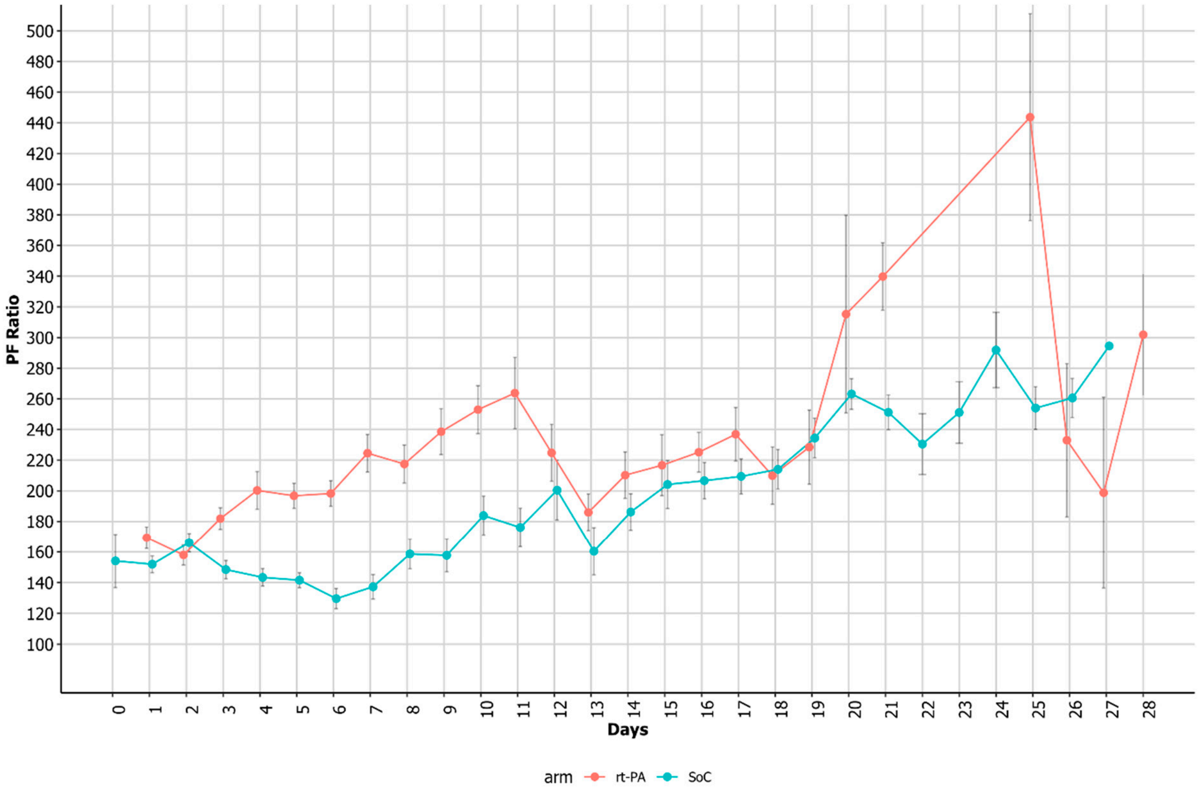

Circles indicate sample mean values (n=9; rt=PA and n=18, SoC). Vertical grey bars indicate the standard error of the corresponding sample means. Time-point 0 is the baseline, all available P/F ratios were extracted and then data was summarised every four hours (± 2h).

P/F, PaO_2/FiO_2; rt-PA, recombinant tissue plasminogen activator; SE, standard error; SOC, standard of care.

Figure S4: Cohort 2 – Lowest daily P/F ratio over time for IMV and NIRS subgroups

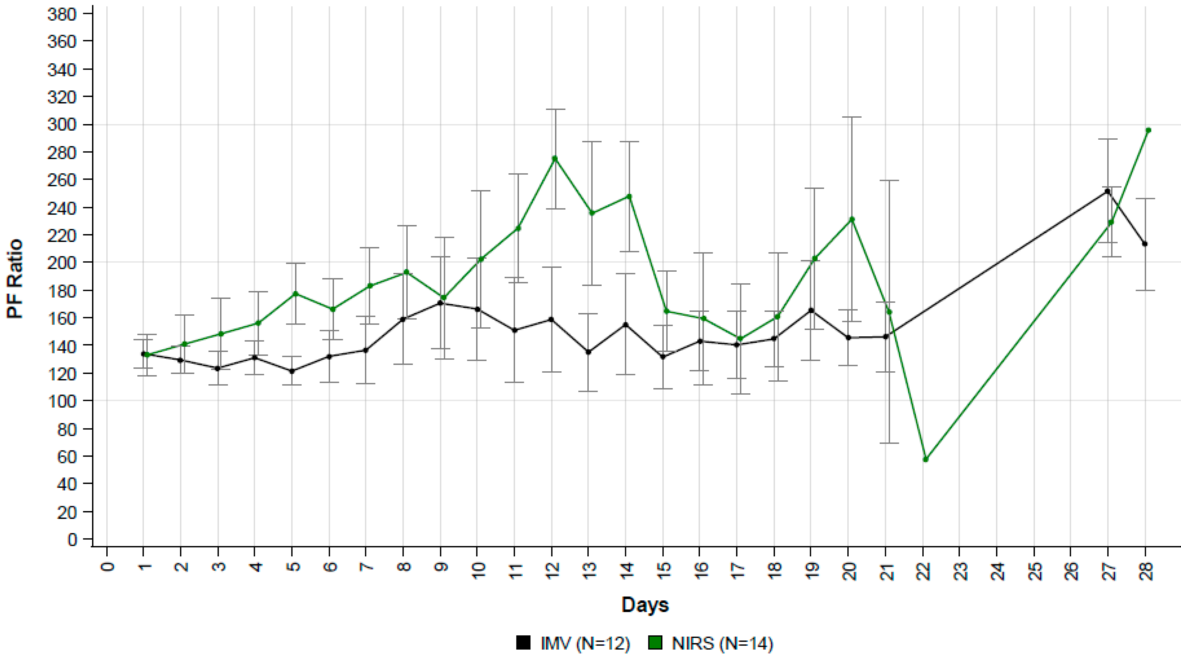

Circles indicate sample mean values (n=12; IMV and n=14, NIRS). Vertical grey bars indicate the standard error of the corresponding sample means. A maximum of six P/F ratios including the lowest ratio over a 24-hour period was extracted, but the graph includes the lowest ratio for the day.

IMV, invasive mechanical ventilation; NIRS, non-invasive respiratory support; P/F, PaO2/FiO2; SE, standard error.

Figure S5. Biomarker activity during rt-PA treatment in Cohort 1, Cohort 2 IMV, and Cohort 2 NIRS.

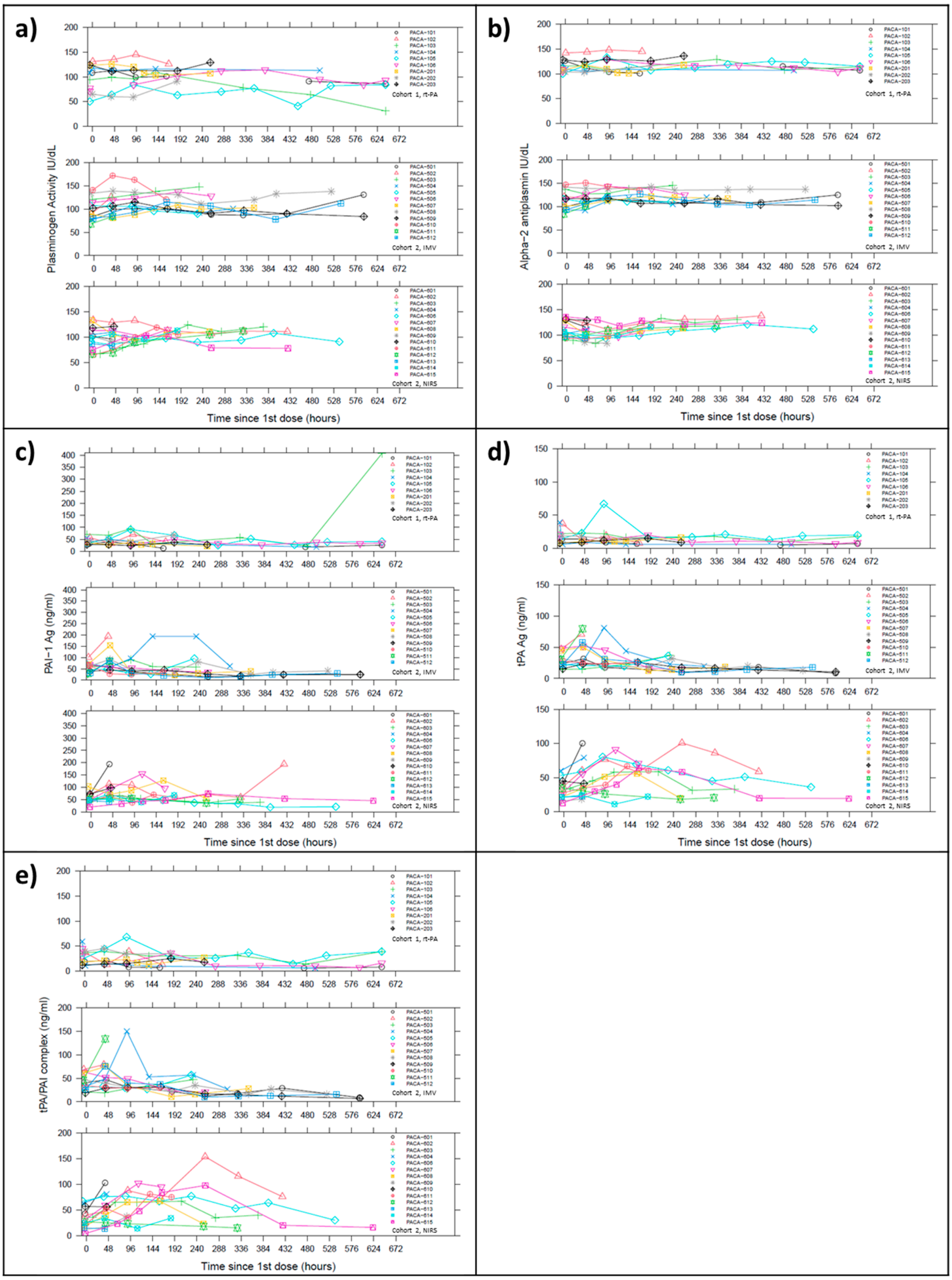

The figure shows the activity of the plasminogen (a), α2AP (b), PAI-1 Ag (c), t-PA Ag (d) and rt-PA/PAI complex (e) biomarkers during rt-PA treatment in Cohort 1 (top) and among Cohort 2 IMV (middle) and Cohort 2 NIRS (bottom) groups (post-hoc exploratory results).

Ag, antigen; α2AP, alpha-2 antiplasmin; IMV, invasive mechanical ventilation; NIRS, non-invasive respiratory support; PAI-1, plasminogen activator inhibitor-1; t-PA, tissue plasminogen activator antigen.
